# Proteomic Profiling Reveals How Physiological Media Reshape Cancer Cell Proteomes and Signaling Networks

**DOI:** 10.1016/j.mcpro.2026.101569

**Published:** 2026-04-15

**Authors:** Colin Zenge, Brittany Q. Pham, Ki Hong Nam, Elana Apfelbaum, Heeseon An, Alban Ordureau

**Affiliations:** 1 Cell Biology Program, Sloan Kettering Institute, Memorial Sloan Kettering Cancer Center, New York, New York, USA; 2 Louis V. Gerstner Jr. Graduate School of Biomedical Sciences, Memorial Sloan Kettering Cancer Center, New York, New York, USA; 3 Department of Pharmacology, Weill Cornell Graduate School of Medical Sciences, New York, New York, USA; 4 Molecular Pharmacology Program, Sloan Kettering Institute, Memorial Sloan Kettering Cancer Center, New York, New York, USA; 5 Chemical Biology Program, Sloan Kettering Institute, Memorial Sloan Kettering Cancer Center, New York, New York, USA; 6 Tri-Institutional PhD Program of Chemical Biology, Memorial Sloan Kettering Cancer Center, New York, New York, USA

**Keywords:** physiological media, proteomics, cancer cell metabolism, mTORC1 signaling, CDK activity

## Abstract

Altered metabolism is a hallmark of cancer, making metabolic enzymes attractive therapeutic targets. However, metabolic inhibitors have shown limited clinical success, partly due to differences between standard culture media and physiological nutrient conditions. Human plasma-like medium (HPLM) better recapitulates *in vivo* metabolite concentrations, yet its effects on cellular proteomes remain poorly characterized. We performed comprehensive TMTpro-based quantitative proteomics and phosphoproteomics across nine cancer cell lines cultured in DMEM or HPLM, consistently quantifying over 10,000 proteins and 24,000 phosphorylation sites across all three biological replicates with high reproducibility. Physiological media induced profound cell-type-specific remodeling of metabolic networks, mitochondrial proteomes, and signaling pathways. While decreased mTORC1 and CDK activity represented universal responses across all cell lines, metabolic enzyme expression exhibited striking heterogeneity. Enzymes in folate metabolism and pyrimidine salvage pathways showed consistent reductions across all cell types, indicating that drug responses may vary with media choice. Mitochondrial proteome composition and morphology displayed cell-type-specific adaptations. Phosphoproteomic analysis revealed kinase signaling networks underlying these metabolic changes. This dataset, accessible via an interactive web application, provides a resource for metabolic research using physiological media, highlighting substantial cell-type-specific variability in how media affect proteomes and signaling pathways.

## Highlights

• Comprehensive proteomic atlas of nine cancer cell lines grown in physiological media.
• Physiological media induce cell-type-specific remodeling of mitochondrial morphology and metabolic and organellar proteomes.
• Reduced proliferation with lower CDK activity is a universal response to media.
• Cell-type-specific metabolic-proteome reshaping may drive drug and stress responses.

## Graphical Abstract

**Figure:**
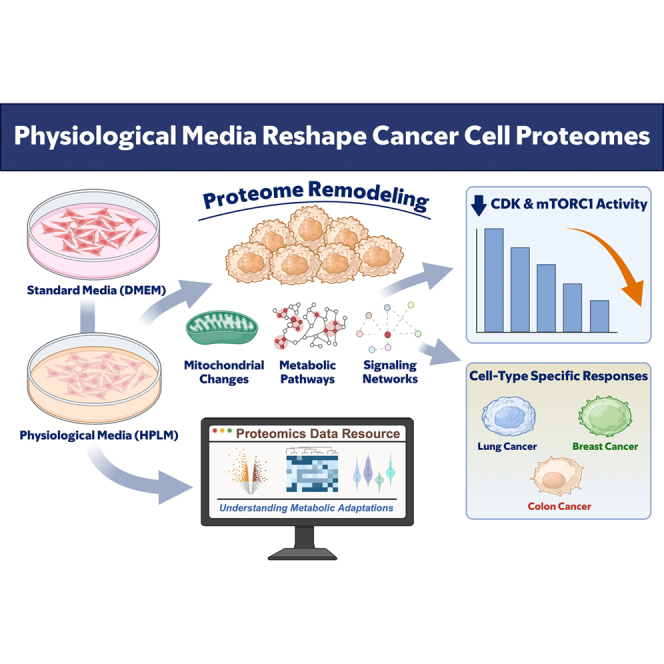

## In Brief

Zenge & Pham *et al*. provide a comprehensive proteomic resource for physiological media research. They systematically profiled nine cancer cell lines cultured in human plasma-like medium, revealing that while some responses are universal (reduced CDK and mTORC1 activities), metabolic proteome remodeling is strikingly cell-type-specific. This atlas highlights critical considerations for the development of metabolic drugs and the selection of cell lines.

Controlled cellular proliferation depends on the precise sensing and integration of intracellular metabolic cues through nutrient-responsive signaling pathways that coordinate cellular growth, metabolism, and fate decisions. For example, AMP-activated protein kinase (AMPK) functions as a key sensor of cellular energy status by monitoring the AMP/ADP-to-ATP ratio and adjusting metabolism in response to energy stress (1). Conversely, cellular nutrient status, such as amino acid levels, is primarily sensed by the mammalian target of rapamycin complex 1 (mTORC1) and general control nonderepressible-2 kinase (GCN2). Of those, mTORC1 is a central regulator that integrates nutrient and growth signals to control biosynthetic and catabolic processes (2, 3, 4). Approximately 70% of cancers exhibit mutations that trigger the hyperactivation of mTORC1, leading to cellular metabolism that sustains cancer growth and proliferation (5).

Metabolites, the small-molecule intermediates and end products of enzymatic reactions (6), participate in a wide range of cellular processes. Beyond enhanced glycolysis, cancer cells exhibit numerous metabolic adaptations, including increased lipogenesis, altered fatty acid oxidation, and changes in serine and glycine metabolism (7, 8). Consequently, numerous metabolic enzymes, including components of the mTORC1 pathway, have attracted interest as drug targets, with several showing promising results in tissue culture models (9, 10, 11). However, many of these small-molecule modulators have shown limited efficacy *in vivo*, underscoring a key limitation of current tissue culture systems: they do not replicate the nutrient environment cells encounter in tissues. One reason for this is that the composition of conventional media differs from what cells actually encounter in a tissue environment (12, 13). Most culture media used today were developed in the 1950s to support the growth of specific cell lines (14, 15, 16). They usually contain higher levels of glucose, glutamine, and pyruvate compared to human serum but lack certain metabolic intermediates or by-products present in serum, such as uric acid and creatine (17). These disparities may affect various metabolic pathways and cellular signaling mechanisms, resulting in divergent responses to their pharmacological inhibition (13, 18).

Recently, two media, human plasma-like medium (HPLM) and Plasmax, have been reported to better mimic the amino acid levels and metabolite profiles of human blood plasma (17, 19). Although developed by independent laboratories, they share similar formulations and are commercialized for broad use by the research community. The use of these media has resulted in intriguing insights regarding how metabolites in the media can impact cellular metabolic enzymes and their essentiality. For example, Cantor *et al*. found that blood cells cultured in HPLM exhibited reduced pyrimidine synthesis, caused by elevated uric acid levels in HPLM, which was reported to inhibit uridine monophosphate synthase (UMPS) activity in pyrimidine *de novo* synthesis (17). This change in UMPS activity was suggested to decrease cellular sensitivity to 5-fluorouracil (5-FU), a pyrimidine analog widely used for anticancer therapy. This study highlights how the choice of media can influence the outcome of chemotherapy. In parallel, Vande Voorde *et al*. found that breast cancer cells grown in Plasmax lacked the hypoxic-like conditions common in cells cultured in DMEM-F12 media due to the high pyruvate levels added to the media, which stabilize hypoxia-inducible factor 1-alpha (HIF-1α) (19). Studies from other labs have reported that cells grown in these media show markedly different metabolic traits, redox states, and glucose utilization, compared to those in traditional media such as RPMI or DMEM-F12, when assessed using both blood cells and epithelial cancer cell lines. Accumulating studies, thus, indicate that the choice of media can influence various aspects of cellular metabolism and beyond (20, 21, 22).

Many alterations caused by these physiological media depend on the cell lines (20). For example, transcriptomic analysis found over a thousand genes with different expression levels between the two media, but only 5% of these genes were shared across two or more cell lines (19). Physiological media also caused morphological changes, the extent of which varied across cell lines (23). At present, the alteration in cellular pathways affected by these media at the global level and the extent of heterogeneity remains unclear. As these media gain traction for metabolism research and show potential to overcome limitations of the current tissue culture system, conducting systematic proteome analyses that compare cells cultured in traditional *versus* physiological media can enhance our understanding and expectations of these new media. These quantitative data will provide a comprehensive resource for proteome remodeling and may identify specific metabolic pathways associated with cell type heterogeneity.

Here, we present a systematic analysis of proteome and phosphoproteome differences across 9 cell lines with various lineages after culturing them in DMEM and a physiological medium, HPLM, in three biological replicates. We selected common laboratory cell lines covering a range of human tissue origins. The study quantifies 9002 proteins, covering ∼80% of the proteome expressed, and 15,149 phosphosites in all three replicates. Data analyses show that the extent of proteome changes varied depending on the cell lines, with breast cancer lines exhibiting the least change, while a cell line with high genomic instability, HCT116, showed the most significant proteome reshaping. Mitochondrial proteins involved in the electron transport chain, especially those in complex I and IV, were significantly reduced in multiple cell lines, along with an increase in phosphorylation of ubiquitin at Serine 65, a marker for mitophagy, in select cell lines. Interestingly, most subcellular organelles showed heterogeneous, bidirectional changes in proteome mass across the 9 cell lines, with ribosomal proteins exhibiting cell-type-specific regulation of the large- and small-subunit balance. Additionally, phosphorylation levels of several ribosomal proteins decreased in HPLM, including those targeted by CDK2 to regulate protein translation during mitosis. Consistent with this, kinase activity prediction indicates reduced CDK activity as a common feature, and we observed lower cell proliferation rates in HPLM. Overall, the HPLM causes global changes in metabolic enzymes, subcellular organelles, and the cell cycle. Additionally, we have created a user-friendly online viewer to facilitate exploration of this extensive dataset (see the "Data and code availability" section). Overall, this study highlights the significant yet varied impact of media, serving as a valuable resource in the field for those using physiological media in metabolic research.

## Experimental Procedures

### Cell Lines

DU145 (human prostate carcinoma, male, ATCC HTB-81, RRID: CVCL_0105), HCT116 (human colorectal carcinoma, male, ATCC CCL-247, RRID: CVCL_0291), HEK293T (human embryonic kidney, fetus, ATCC CCL-3216, RRID: CVCL_0063), HeLa (human uterine adenocarcinoma, female, ATCC CRM-CCL-2, RRID: CVCL_0030), MCF7 (human breast adenocarcinoma, female, ATCC HTB-22, RRID: CVCL_0031), PANC-1(human pancreatic adenocarcinoma, male, ATCC CRL-1469), PC-3 (human prostate adenocarcinoma, male, ATCC CRL-1435, RRID: CVCL_0035), hTERT RPE-1 (human retinal pigment epithelium, ATCC CRL-4000, RRID: CVCL_4388), and U2OS (human osteosarcoma, female, ATCC HTB-96, RRID: CVCL_0042) cells were grown in either Dulbecco’s modified Eagle’s medium (DMEM, high glucose and pyruvate (Gibco)) supplemented with 10% dialyzed fetal bovine serum (Gibco, US origin) or HPLM media (Gibco) and 10% dialyzed fetal bovine serum (Gibco, US origin) for ∼15 days (over three passages) prior to the proteomic analyses. This duration was chosen based on protein turnover kinetics: assuming approximately 50% of the proteome is replaced per cell division, 5 to 6 divisions are required to replace more than 98% of the original protein pool. All the cells were maintained in a 5% CO_2_ incubator at 37 °C.

### Antibodies and Chemicals

The following antibodies and reagents were used in this study: HMGCS1 (36877S, Cell Signaling Technology), HMGCR (Ab242315, Abcam), phospho-S6 ribosomal protein Ser235,236 (4858S, Cell Signaling Technology), Tubulin (ab7291, Abcam), pT160 CDK2 (Cell Signaling Technology, 2561S), CDK2 (ProteinTech Group, 10122-1-AP), pS6K (Cell Signaling Technology, 9234S), 4EBP1 (Cell Signaling Technology, 9644T), RhoA (Cell Signaling Technology, 2117T), UMPS (ProteinTech Group, 14830-1-AP), UCK2 (ProteinTech Group, 10511-1-AP), DHODH (ProteinTech Group, 14877-1-AP), IRDye 800CW Goat anti-Rabbit IgG H + L (926–32211, LI-COR), IRDye 800CW Goat anti-Mouse IgG H + L (926–32210, LI-COR), IRDye 680 RD Goat anti-Mouse IgG H + L (926–68070, LI-COR), Benzonase Nuclease HC (71,205–3, Millipore), REVERT Total Protein Stain kit (LI-COR, P/N926–11010), sodium dodecyl sulfate (SDS) (Fisher Scientific, PI28364), chloroacetamide (Fisher Scientific, AAA1523830), TCEP (Gold Biotechnology), Formic acid (Sigma Aldrich, 94,318), TMTpro 18plex Label Reagent (Fisher Scientific, A52045), and InstantBlue (Abcam, ab119211).

### Cell Lysis and Immunoblotting Assay

Cells were grown in DMEM or HPLM for ∼15 days, and cell confluency did not exceed 70% at the time of harvest. Cell pellets were lysed with in-house RIPA buffer (50 mM HEPES, 150 mM NaCl, pH 7.6, 1% NP-40, 1% sodium deoxycholate, 0.1% SDS, 10 mM glycerophosphate, 10 mM sodium pyrophosphate, protease inhibitor cocktail, phosphatase inhibitor cocktail) containing 2 mM TCEP, 15 mM MgCl_2_, and benzonase (Millipore, for RIPA only). Lysates in RIPA buffer were then sonicated, and a Bradford assay was performed to measure the protein concentration. The normalized cell lysates were denatured by adding lithium dodecyl sulfate (LDS) supplemented with 50 mM DTT, followed by boiling at 90°C for 5 min. Less than 30 μg of each lysate was loaded onto the 4 to 12% NuPAGE Bis-Tris gel (Thermo Fisher Scientific), followed by SDS-PAGE with MES (Thermo Fisher Scientific) running buffer. Following transfer to PVDF membranes (0.45 μm or 0.2 μm, Millipore), blocking was performed with 5% non-fat milk at room temperature for 15 min, then incubated with primary antibodies diluted in TBST containing 2% BSA (1:1000) overnight at 4 °C. After the incubation with primary antibodies, membranes were washed with TBST and further incubated with fluorescent IRDye antibody (1:20,000; LICORbio) for 1 h at room temperature. The membrane was then imaged using Chemidoc MP (Bio-Rad) or Odyssey CLX.

### Proteomics—Sample Preparation and TMTpro Labeling

The total proteomics analyses were performed based on the previously reported method (24). Briefly, all 9 cell lines were plated in two 10 cm dishes, one in DMEM and one in HPLM, for a total of 18 dishes. Cells were then washed with ice-cold PBS five times and lysed with 800 μl of RIPA buffer (50 mM HEPES, 150 mM NaCl, pH 7.6, 1% NP-40, 1% sodium deoxycholate, 0.1% SDS, 10 mM glycerophosphate, 10 mM sodium pyrophosphate, protease inhibitor cocktail, phosphatase inhibitor cocktail, pH 7.5). The lysate was then collected and sonicated three times, followed by Bradford assay to measure the protein concentration. The total protein concentration was adjusted to 3 mg/ml across all samples using RIPA buffer. 125 μg of each protein extract was reduced by incubation with 5 mM TCEP (MS-grade, Sigma Aldrich) at 55 °C for 10 min. After cooling to room temperature, the lysates were reacted with a chloroacetamide solution (final conc. 20 mM, Sigma Aldrich) for 15 min, followed by chloroform/methanol precipitation. Protein discs were resuspended in 100 mM EPPS (4-(2-Hydroxyethyl)-1-piperazinepropanesulfonic acid, Thermo Fisher Scientific) (pH 8.5) containing 0.1% RapiGest (Waters) and digested at 37 °C overnight with MS-grade Trypsin (100:1 protein-to-protease ratio, Thermo Fisher Scientific). 18-plex tandem mass tag (Thermo Fisher Scientific) labeling of each sample was performed by adding 10 μl of the 20 ng/ml stock of TMTpro reagent, along with acetonitrile, to achieve a final acetonitrile concentration of approximately 30% (v/v). Following incubation at room temperature for 1 h, the digestion and labeling efficiency of a small aliquot was tested. The reaction was then quenched with hydroxylamine (Sigma Aldrich) to a final concentration of 0.5% (v/v) for 15 min. The TMTpro-labeled samples were pooled together at a 1:1 ratio. The sample was vacuum centrifuged to near dryness and subjected to C18 solid-phase extraction (SPE) (100 mg, Sep-Pak, Waters).

### Proteomics - Fe^3+^-NTA Phosphopeptide Enrichment

Phosphopeptides were enriched using the Pierce High-Select Fe^3+^-NTA phosphopeptide enrichment kit (Thermo Fisher Scientific, A32992) following the provided protocol. In brief, dried peptides were enriched for phosphopeptides, eluted into a tube containing 25 μl of 10% formic acid to neutralize the pH of the elution buffer, and dried down. The unbound peptides (flow-through) and washes were combined and saved for total proteome analysis.

### Proteomics—Offline Basic pH Reversed-phase Fractionation

For Phospho-peptides, the dried TMTpro-labeled peptides were fractionated according to the manufacturer’s instructions using the High pH reversed-phase peptide fractionation kit (Thermo Fisher Scientific, 84,868) for a final six fractions and subjected to C18 StageTip desalting (Empore C18 SPE Disk) before MS analysis.

For non-phosphorylated peptides, the dried TMTpro-labeled sample was resuspended in 100 μl of 10 mM NH_4_HCO_3_ (pH 8.0, Sigma Aldrich) and fractionated using basic pH reverse-phase HPLC (25). Briefly, samples were off-line fractionated over a 90 min run, into 96 fractions by high pH reverse-phase HPLC (Agilent LC1260 Infinity II, equipped with a degasser and wavelength detector (set at 214 nm)) through an aeris peptide xb-c18 column (Phenomenex), with mobile phase A containing 5% acetonitrile and 10 mM NH_4_HCO_3_ in LC-MS grade H_2_O, and mobile phase B containing 90% acetonitrile and 10 mM NH_4_HCO_3_ in LC-MS grade H_2_O (both pH 8.0). The 96 resulting fractions were then pooled in a non-continuous manner (26) into 24 fractions (whole proteome). Fractions were vacuum centrifuged to near dryness. Each consolidated fraction was desalted using a StageTip (Empore C18 SPE Disk), dried again by vacuum centrifugation, and reconstituted in 5% acetonitrile, 1% formic acid for MS analysis.

### Proteomics—Total Proteomics Analysis Using TMTpro

Mass spectrometry data were collected using an Orbitrap Eclipse Tribrid mass spectrometer coupled to an UltiMate 3000 RSLCnano system liquid chromatography (LC) pump. Peptides were separated on a 100 μm inner diameter microcapillary column packed in-house with ∼40 cm of HALO Peptide ES-C18 resin (2.7 μm, 160 Å, Advanced Materials Technology, Wilmington, DE) with a gradient consisting of 5%–21% (0–85 min), 21 to 28% (85–110 min) (acetonitrile, 0.1% FA) over a total 125 min run at ∼500 nl/min. For analysis, we loaded 1/10 of each fraction onto the column. Each analysis used the Multi-Notch MS (3)-based TMT method (27), to reduce ion interference compared to MS (2) quantification (28), combined with the FAIMS Pro Interface (using previously optimized 3 CV parameters for TMT-multiplexed samples (29)) and combined with the newly implemented Real-Time Search analysis software (30, 31). The scan sequence began with an MS (1) spectrum (Orbitrap analysis; resolution 120,000 at 200 Th; mass range 400 − 1500 m/z; automatic gain control (AGC) target 4 × 10^5^; maximum injection time 50 ms). Precursors for MS (2) analysis were selected using a 1.25 s/CV cycle type (FAIMS, CV = - 40/-60/-80). MS^2^ analysis consisted of collision-induced dissociation (quadrupole ion trap analysis; Rapid scan rate; AGC 1.0 × 10^4^; isolation window 0.5 Th; normalized collision energy (NCE) 35; maximum injection time 35 ms). Monoisotopic peak assignment was used, and previously interrogated precursors were excluded using a dynamic window (180 s ± 10 ppm). Following the acquisition of each MS (2) spectrum, a synchronous-precursor-selection (SPS) API-MS (3) scan was collected on the top 10 most intense ions b or y-ions matched by the online search algorithm in the associated MS (2) spectrum (30, 31). MS (3) precursors were fragmented by high-energy collision-induced dissociation (HCD) and analyzed using the Orbitrap (NCE 45; AGC 2.5 × 10^5^; maximum injection time 200 ms, resolution was 50,000 at 200 Th). The closeout was set to two peptides per protein per fraction, so MS^3^s were no longer collected for proteins with two peptide-spectrum matches (PSMs) that passed the quality filters.

### Proteomics: Phosphoproteomics Analysis Using TMTpro

Mass spectrometry data were collected using an Orbitrap Eclipse Tribrid mass spectrometer coupled to an UltiMate 3000 RSLCnano system liquid chromatography (LC) pump. Peptides were separated on a 100 μm inner diameter microcapillary column packed in-house with ∼40 cm of HALO Peptide ES-C18 resin (2.7 μm, 160 Å, Advanced Materials Technology) with a gradient of acetonitrile (0.1% FA) over a total 180 min run at ∼500 nl/min. For analysis, we loaded half of each fraction onto the column. Each analysis used the FAIMS Pro Interface (using previously optimized 3 CV parameters for TMTpro-labeled phosphopeptides (32)) to reduce ion interference. The scan sequence began with an MS (1) spectrum (Orbitrap analysis; resolution 120,000 at 200 Th; mass range 400 − 1500 m/z; automatic gain control (AGC) target 6 × 10^5^; maximum injection time 50 ms). Precursors for MS (2) analysis were selected using a 1.25 s/CV cycle type (FAIMS, CV = - 40/-60/-80). MS^2^ analysis consisted of high-energy collision-induced dissociation (HCD) (Orbitrap analysis; resolution 50,000 at 200 Th; isolation window 0.5 Th; normalized collision energy (NCE) 38; AGC 2 × 10^5^; maximum injection time 172 ms). Monoisotopic peak assignment was used, and previously interrogated precursors were excluded using a dynamic window (150 s ± 10 ppm).

### Proteomics: Data analysis

Mass spectra were converted to mzXML (33) and processed using the open-source Comet search engine (2020.01 rev. 4) software pipeline (34) with the Human Reference Proteome (2020–03 - SwissProt entries only, containing 20,353 canonical and 21,992 isoform entries), UniProt database (35) with contaminants, sorted by length, and reverse decoy sequences appended. Searches were performed with a 50-ppm precursor ion tolerance using trypsin, allowing up to two miscleavages. The minimum and maximum peptide lengths for analysis were set to 7 and 63 residues, respectively. For total proteomic analysis, the recommended product ion parameters for ion traps were used (tolerance = 1.0005, offset = 0.4 (mono masses), theoretical fragment ions = 1). For phosphoproteomics analysis, the recommended product ion parameters for high-resolution were used (0.02 tolerance, 0.0 offset (mono masses), theoretical fragment ions = 0). TMTpro tags on lysine residues and peptide N termini (+304.207 Da) and carbamidomethylation of cysteine residues (+57.021 Da) were set as static modifications, while oxidation of methionine residues (+15.995 Da) was set as a variable modification. For the phosphorylation dataset search, phosphorylation (+79.966 Da) on Serine, Threonine, or Tyrosine, and deamidation (+0.9840 Da) on asparagine and glutamine were set as additional variable modifications. For any fragment ion phosphorylation modification, a neutral loss of −97.9769 Da was also analyzed. Peptide-spectrum matches (PSMs) for each run were adjusted to a 1% false discovery rate (FDR) (36). PSM filtering was performed using a linear discriminant analysis employing a target-decoy strategy (37), while considering the following parameters: Comet Log Expect, ΔCn, charge state, missed cleavages, fraction of ions matched, precursor mass accuracy, and peptide length. Filtered PSMs were then further collapsed using the Picked FDR method (38) to achieve a target FDR of 1%. Moreover, protein assembly was guided by principles of parsimony to produce the smallest set of proteins necessary to account for all observed peptides. For TMTpro-based reporter ion quantitation, we extracted the summed signal-to-noise (S:N) ratio for each TMTpro channel and found the closest matching centroid to the expected mass of the TMT reporter ion (integration tolerance of 0.003 Da). Reporter ion intensities were adjusted to correct for the isotopic impurities in the different TMTpro reagents, as specified by the manufacturer. Proteins were quantified by summing reporter ion signal-to-noise measurements across all matching PSMs, yielding a ‘‘summed signal-to-noise’’ measurement. For the total proteome, PSMs with poor quality, MS (3) spectra with up to 17 TMTpro reporter ion channels missing, isolation specificity below 0.7, a TMTpro reporter summed signal-to-noise ratio less than 180, or no MS^3^ spectra were excluded from quantification. For the phosphoproteome, PSMs with poor quality, spectra with up to 17 TMTpro reporter ion channels missing, isolation specificity below 0.75, or TMTpro reporter summed signal-to-noise ratio under 135 were excluded from quantification. Phosphorylation site localization was determined using the AScorePro algorithm (39, 40). AScore is a probability-based approach for high-throughput localization of protein phosphorylation sites. Specifically, a threshold of 13 corresponded to 95% confidence in site localization.

Protein or peptide quantification values were exported for further analysis in Microsoft Excel, GraphPad Prism, JMP software, R in RStudio, clusterProfiler (41), and Perseus (42). For each set (independent plex), each reporter ion channel was summed across all quantified proteins and then normalized, assuming equal protein loading in all samples. The TMT signal was scaled to 180 (i.e., 10 per channel) for each set, and a maximum fold change of 100 was allowed. No normalization was performed between different plexes. Phosphopeptides were first normalized to the corresponding protein abundance value (when available). The dataset was then normalized and PCA was performed using the PhosR package with a k-value parameter of three (optimal value when benchmarked against other values) (43). Kinase prediction was performed using the https://kinase-library.mit.edu/home (44). Foreground definition for kinase prediction was set to log fold change threshold of 1, *p*-value threshold of 0.05, and score percentile rank threshold of 15. Only fully localized, non-composite serine or threonine phosphosites were chosen for analysis. Transcription factor enrichment analysis was performed using ChEA3 (45).

### Flow Cytometry Analysis

Cells were plated in 6-well plates in triplicate per condition and treated for 16 h with the respective inhibitors. Samples were harvested using trypsin (Gibco), resuspended in sorting buffer (1X DPBS, 1 mM EDTA, 25 mM HEPES, 1% FBS, final pH 7.3–7.5), and filtered through 35 μm cell strainer caps. Flow cytometry was performed on either the FACS Aria (BD Biosciences) or the SONY SH800 (Sony Biotechnology), and analysis was done using the FlowJo software. The distribution graph was processed in Prism software after exporting the individual cells’ intensity values.

### Cell Proliferation Assay

Cells were plated in 24-well plates in triplicate the day before each time point (Day 0–3, 4–7, 8–11, and 12–15) in either HPLM or DMEM. Proliferation curves were generated by live-cell imaging on the Sartorius IncuCyte S3 (37 °C with 5% CO_2_) every 12 h for the 3-day experiment window. Four brightfield 10X images per well were acquired at every time point. Percent confluency analysis was performed using the IncuCyte software's integrated cell-by-cell algorithms and averaged across each time point.

### Mito-Keima Assay

PC3 and PANC1 cell lines were infected with lentivirus encoding for a mitochondrial matrix-targeted mKeima construct (mtx-mKeima^XL^) (46) and selected with puromycin (1 μg/ml). Cells were then FACS-sorted into a uniform population (intensity) for the Mito-Keima, cultured in either DMEM or HPLM for 15 days, and analyzed by flow cytometry. As a positive control, the mitochondrial depolarizing agents antimycin (10 μM) and oligomycin (5 μM) were added to DMEM-cultured cells for 6 h. Cells were harvested by trypsin, resuspended in sorting buffer (1X DPBS, 1 mM EDTA, 25 mM HEPES, 1% FBS, final pH 7.3–7.5), and filtered through 35 μm strainer caps. Cells were analyzed by flow cytometry on the FACS Aria (BD Biosciences). The data was processed by FlowJo software, and the red-to-green ratio was calculated and exported into Prism software for plotting.

### Live-Cell Confocal Microscopy and Analysis

48 h prior to the imaging analysis, cells were plated onto 33 mm glass bottom flasks (No. 1.5, 14 mm glass diameter, MatTek) pre-treated with poly-L-lysine (Sigma Aldrich) and incubated in the corresponding media for 48 h. For MitoTracker assay, MitoTracker Deep Red (MedChem Express) (0.5 μM) was added to the cells 30 min prior to the imaging. Right before the imaging, the media was replaced with pre-warmed phenol-red-free medium (FluoroBrite DMEM, Thermo Fisher) supplemented with 10% FBS. The cells were imaged using a Yokogawa spinning disk confocal on an inverted Nikon Ti fluorescence microscope equipped with a Plan Apo TIRF 60 × /1.49 N A. objective lens (oil immersion) with SoRa mode for super-resolution imaging. Laser intensity and exposure time were optimized to prevent photobleaching. Nikon NIS-element software was used to denoise and deconvolute the image, and the images were further processed using Fiji.

### Experimental Design and Statistical Rationale

In our study, we examined nine distinct cell lines in a comprehensive comparative TMTpro proteomics analysis exploring proteome remodeling during adaptation to physiological media (HPLM). Each cell line included three biological replicates for HPLM-cultured cells and three for DMEM-cultured controls, totaling 54 samples analyzed across three independent 18plex experiments. For downstream analysis, two types of statistical tests were performed: a two-way analysis of variance (ANOVA) on the entire dataset and a set of parametric, two-sample independent (unpaired) t-tests (Welch’s *t* test) for each cell line. *p*-values in the volcano plots were calculated using two-sided Welch’s *t* test with adjustment for multiple comparisons (details in each Supplemental Table), followed by a strict cutoff for graphical representation at -log_10_(*p*-value) of two (*p* < 0.01). Initial analysis using static parameters (e.g., 'Class A′ with s0 = 0.1 and 1% FDR, or “Class B” with s0 = 0.1 and 5% FDR) revealed that the 9 cell lines separated into two distinct groups: 5 cell lines displayed extensive (50% or more) proteome remodeling while four showed more modest (18% or less) changes (Supplemental Table S1). This observed disparity when using a uniform parametric two-sample inferential test made it difficult to directly compare all 9 cell lines. To facilitate downstream analysis, we balanced the S0 parameter between these two groups, while maintaining a fixed FDR (details in each Supplemental Table S1). A two-way ANOVA was conducted using Perseus software, with cell type and media conditions as the two categorical independent variables (results in Supplemental Tables S1 and S2). The resulting ANOVA *p*-values were adjusted to q-values using a two-stage step-up method of Benjamini, Krieger, and Yekutieli (Prism software) to control the false discovery rate (FDR) at 0.01% for the proteome and 1% for the phosphoproteome. Quantified data comparisons were performed by unpaired Student’s *t* test; Statistical significance thresholds: ∗*p*< 0.05, ∗∗*p*< 0.01, ∗∗∗*p*< 0.001, ∗∗∗∗*p*< 0.0001.

## Results

### Systematic Analysis of Proteomic Changes Induced by HPLM

**Fig. 1:**
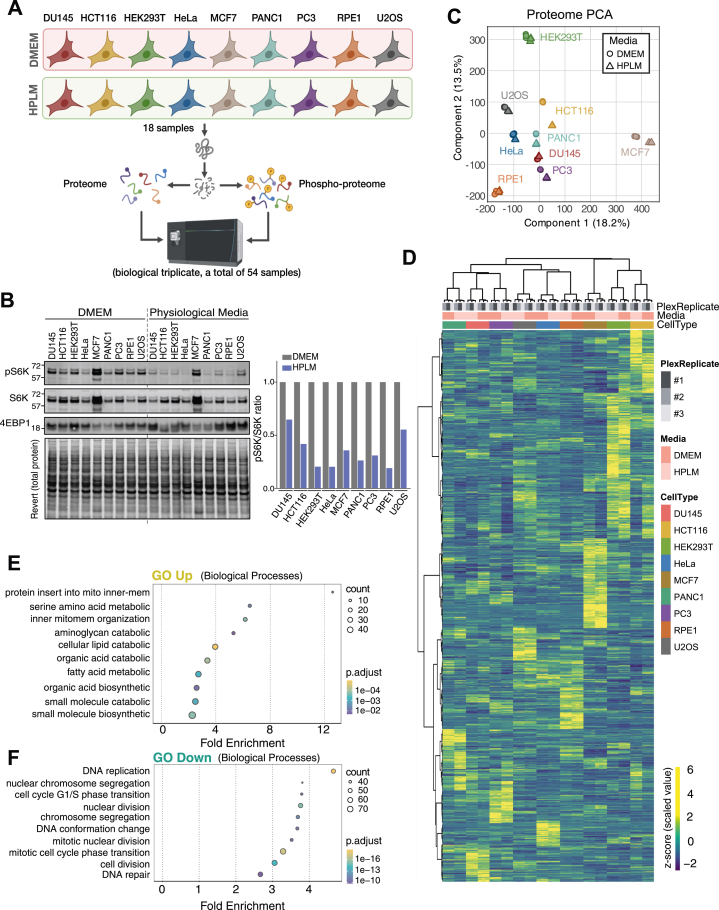
**Systematic analysis of proteomic changes induced by HPLM**. *A*, schematic showing the proteome remodeling of 9 cell lines upon Human Plasma-Like Medium (HPLM) adaptation. *B*, quality control immunoblotting analysis of the 9 cell lines cultured as in panel A shows the overall decrease in mTORC1 activity. The *left panel* shows pS6K quantification. *C*, principal component analysis of the global proteomic dataset, which includes 54 samples from 18 samples across three biological replicates collected on different days, shows tight clustering of the replicates. *D*, heatmap representation of the proteomics data. Proteins’ scaled abundance, transformed into z-scores, significantly contributing to cell type differences based on an ANOVA test (1% FDR corrected), are plotted. *E* and *F*, gene ontology analysis (biological process) of proteins significant by two-way ANOVA for media effect and consistently changed across all 9 cell lines (upregulated ≥0.585 or downregulated ≤ −0.585 median log_2_ fold change) reveals enrichment of metabolic and catabolic terms among upregulated proteins (*E*), and DNA replication and mitotic terms among downregulated proteins (*F*), reflecting broad remodeling of the metabolic proteome alongside suppression of cell cycle activity.

We examined how HPLM culture influences the rewiring of the proteome and phosphoproteome by comparing 9 cell lines from different lineages after culturing them in DMEM or HPLM for several passages (∼15 days). To represent diversity, we chose prostate cancer cells (DU145, PC3), colon cancer cells (HCT116), cervical cancer cells (HeLa), breast cancer cells (MCF7), osteosarcoma cells (U2OS), hTERT-immortalized retinal pigment epithelial cells (RPE-1), and epithelial-like kidney cells (HEK293T) that are widely used in cell biological research (Fig. 1*A*). The 9 cell lines demonstrated varying morphological changes upon HPLM culture (Supplemental Fig. S1*A*). DU145, HeLa, RPE1, and U2OS showed no notable differences between the two media, whereas the other cell lines experienced significant morphological alterations and grew more slowly. A prior study reported that U2OS cells grown in HPLM showed reduced mTORC1 activity (47). Consistently, immunoblot analysis showed significantly reduced phospho-S6K levels in all 9 cell lines under HPLM conditions, suggesting that a downregulation of mTORC1 activity is a common feature of HPLM-grown cells (Fig. 1*B*).

Using 18-plex tandem-mass-tag (TMTpro) across three sets of independent biological replicates (54 samples total), we quantified 11,482 proteins, and over 24,000 phosphorylation sites. Of these, 9002 proteins and 15,149 phosphosites were consistently detected and quantified across all sets (Supplemental Fig. S2, *A* and *B* and Supplemental Table S1–S2). Inter-set correlation was high (Pearson r = 0.95, CV = 5.7%), and principal component analysis (PCA) confirmed tight clustering of replicates within each condition (Fig. 1*C* and Supplemental Fig. S2, *C*–*E*). Cell lines cultured in the two different media grouped together and showed the highest pairwise correlations (Supplemental Fig. S2*C*), indicating that cell identity remains the dominant source of proteome variation, with media-induced changes representing a secondary but substantial component. MCF7 and HEK293T were most distinct from other lines along PC1 and PC2, respectively. Hierarchical clustering of cell lines further reflected the steady-state proteome differences across the 9 cell lines and their response to HPLM culture (Fig. 1*D*). Gene ontology analysis of the commonly upregulated biological processes in HPLM media (two-way ANOVA, media effect) revealed an enrichment of protein insertion into the mitochondrial inner membrane and several other catabolic or metabolic activities, indicating alterations in metabolic proteome (Figs. 1*E* and S3*A*, Supplemental Table S1). Conversely, cell cycle and DNA replication terms, which are involved in cell division, were significantly downregulated, indicating that HPLM treatment reduced overall proliferation, consistent with our immunoblotting analysis on mTORC1 signaling (Figs. 1*F* and S3*B*). Cell line-specific two-sample Welch’s *t* test comparing HPLM vs DMEM culture (see the Experimental Design and Statistical Rationale section) revealed broad and distinct proteome remodeling, with a minimal set of proteins commonly regulated across all cell lines (two proteins), and about a hundred proteins commonly regulated in at least six of the cell lines (69 proteins) (Supplemental Fig. S2*E*, Supplemental Table S1). These two universally regulated proteins are MAT2A, the primary S-adenosylmethionine (SAM) synthase, and PCK2, the mitochondrial phosphoenolpyruvate carboxykinase, whose induction likely reflects direct adaptation to the lower methionine and glucose concentrations in HPLM relative to DMEM, respectively. The broader set of commonly regulated proteins exhibited enrichment of methylation, mitotic cell cycle, and amino acid and one-carbon metabolic terms (Supplemental Fig. S2*F*). Among the most consistently depleted proteins were SAM-dependent methyltransferases spanning diverse substrate classes, including METTL2B (tRNA methyltransferase, significant in 8/9 cell lines), METTL5 (rRNA), NTMT1 (protein N-termini), SETD6, SMYD2, DOT1L, and EZH2 (histones), while arginine methyltransferases PRMT1 and PRMT5 remained unchanged, suggesting a selective suppression of SAM-dependent lysine and non-histone methyltransferases under physiological methionine concentrations. We then analyzed transcription factor networks to identify regulatory circuits that may contribute to HPLM-dependent proteome remodeling. By conducting a transcription factor target over-representation analysis of HPLM-induced differentially expressed proteins (ANOVA, media effect), we identified several transcription factors and transcription factor interactors, with CEBPB as a top-predicted activator and DNMT1 as a top-predicted repressor (Supplemental Fig. S3, *C* and *D*). Examination of these transcription factors showed that CEBPB protein levels were consistently upregulated while DNMT1 protein levels were consistently downregulated across the 9 cell lines (Supplemental Fig. S3, *E* and *F*). While these changes in protein abundance are consistent with the predicted activation or repression of these transcription factors, direct functional validation would be required to confirm alterations in their transcriptional activity. Among consistently regulated proteins across multiple cell lines, the functionally uncharacterized DERPC showed the strongest upregulation (approximately 6-fold mean increase, significant in seven of nine cell lines), while the vitamin B12 trafficking protein MMADHC and the taurine transporter SLC6A6 were consistently downregulated, the latter potentially reflecting reduced import demand when taurine is supplied by HPLM.

### HPLM Culture Induces Heterogeneous Adaptations in the Global, Metabolic, and Organellar Proteomes

**Fig. 2:**
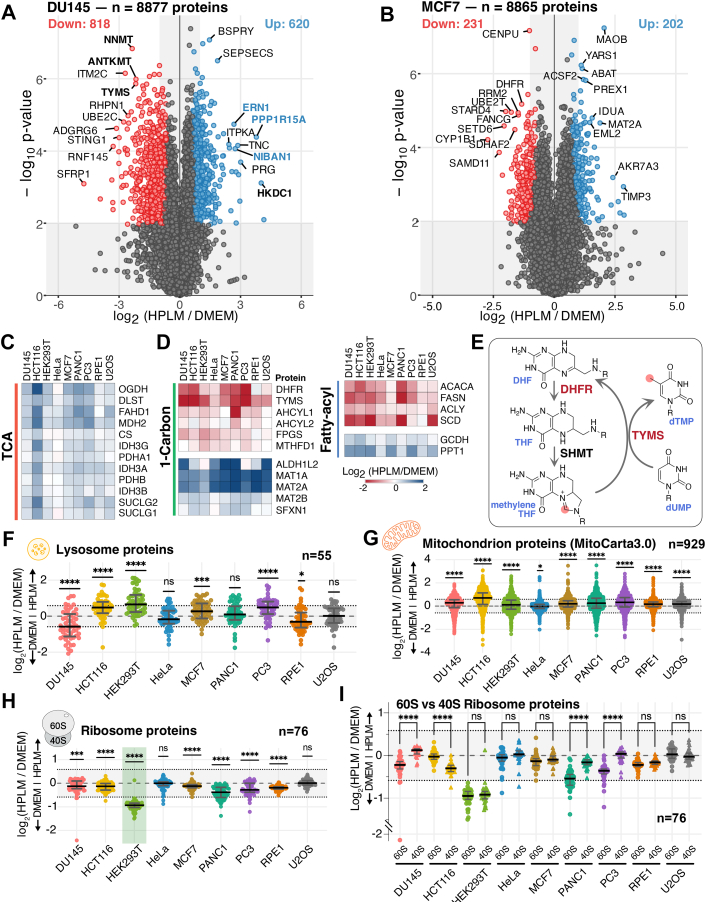
**HPLM culture induces heterogeneous adaptations in the global and organelle proteomes**. *A* and *B*, analysis of the TMTpro-plex data is presented as a volcano plot of the -log10-transformed *p*-value *versus* the log2-transformed ratio of HPLM/DMEM conditions for DU145, panel *A*, and MCF7, panel *B*. *p*-values were calculated using a two-sided Welch’s *t* test (adjusted to 2% FDR for multiple comparisons, S0 = 0.585 for DU145 and 2% FDR, S0 = 0.39 for MCF7). Among the statistically significant hits, proteins that are significantly upregulated are circled in *blue*, while those that are downregulated are in *red*. Only values below *p*-value of 0.01 were considered significant. A total of 8877 proteins were quantified for DU145, and 8865 for MCF7. n = 3 biological replicates. *C*, heatmap of select metabolic enzymes in the tricarboxylic acid cycle (TCA) shows general downregulation across the 9 cell lines tested. The mean of the three biological replicates is used for the heatmap. *D*, some of the 1-Carbon metabolism and fatty-acyl pathway enzymes show a consistent increase across the 9 cell lines, whereas some show a significant depletion. The mean of the three biological replicates is used for the heatmap. *E*, schematic for dTMP biosynthesis from dUMP using methylene THF. The structures of key folate derivatives involved in thymidine synthesis and their respective enzymes are shown. *F*, the scatter plot shows the Log_2_ ratio of HPLM/DMEM for lysosome-localized proteins across the nine cell lines. *Thick black lines* represent the median ratio values (± interquartile range) of 55 representative proteins (n = 3 biological triplicate experiments). *p*-values were calculated using a Wilcoxon signed-rank test to determine whether the differences between the two culture media were statistically significant (α = 0.01). The *grey shaded* area between the dotted lines indicates a ±1.5 ratio change. *G*, the scatter plot shows the Log_2_ ratio of HPLM/DMEM for mitochondrial proteins across the nine cell lines. *Thick black lines* represent the median ratio values (± interquartile range) of 929 representative proteins (n = 3 biological triplicate experiments). *p*-values were calculated using a Wilcoxon signed-rank test to determine whether the differences between the two culture media were statistically significant (α = 0.01). The *grey shaded* area between the *dotted lines* indicates a ±1.5 ratio change. *H*, the scatter plot shows the Log_2_ ratio of HPLM/DMEM for ribosomal proteins across the nine cell lines. *Thick black lines* represent the median ratio values (± interquartile range) of 76 representative proteins (n = 3 biological triplicate experiments). *p*-values were calculated using a Wilcoxon signed-rank test to determine whether the differences between the two culture media were statistically significant (α = 0.01). The *grey shaded* area between the *dotted lines* indicates a ±1.5 ratio change. *I*, As in *H*, but the ribosomal proteins were categorized based on whether they are part of the 60S large subunit or the 40S small subunit. *p*-values were calculated using a Kruskal-Wallis test, with Dunn’s multiple comparison correction to determine if the differences between the two ribosomal subunits were statistically significant (α = 0.01). The *grey shaded* area between the *dotted lines* indicates a ±1.5 ratio change.

The extent of HPLM-induced proteome changes (Welch’s *t* test with 2% FDR correction) varied across the cell types (Supplemental Fig. S2*E*, upper panel). Breast carcinoma cell line MCF7 (4.8% significant hits) and cervical cancer line HeLa (2.4%), along with RPE1 (3.5%) cells, exhibited relatively smaller proteome changes. Conversely, prostate cancer lines DU145 (16%) and PC3 (19.5%), along with colon cancer line HCT116 (26.2%), showed the most dramatic proteome alterations (Figs. 2, *A* and *B*, and S4*A*). Interestingly, the level of global proteome change did not match the degree of morphological alterations. For instance, DU145 exhibited almost no visible change in morphology, yet about 16% of its proteome was significantly altered. Conversely, MCF7 cells, which displayed notable morphological differences after several passages in HPLM culture, showed just 4.8% significant changes in their global proteome.

In each cell line, several metabolic enzymes were identified as proteins with the most significant changes during HPLM culture. In DU145 cells, nicotinamide N-methyltransferase (NNMT) and thymidylate synthase (TYMS) were significantly downregulated, whereas Hexokinase Domain Containing 1 (HKDC1) increased significantly (Fig. 2*A*). Besides metabolic enzymes, stress response proteins also showed significant alterations. For example, among the top 10 hits in DU145, ERN1 (IRE1), NIBAN1, and PPP1R15A (GADD34) are involved in sensing and responding to endoplasmic reticulum (ER) stress. More broadly, these proteins are part of a coherent integrated stress response (ISR) signature observed across multiple cell lines: canonical ATF4 transcriptional targets, including ASNS, SESN2, PSAT1, and PHGDH were consistently upregulated, indicating activation of the GCN2-ATF4 axis in response to the reduced amino acid availability in HPLM.

A key benefit of our extensive multiplexed proteomic data is that we quantified 9002 proteins across 9 cell lines and replicates, which enables us to identify both common and cell-type-specific proteome variations. Using this wealth of data, we examined changes in metabolic enzymes involved in the TCA cycle, one-carbon metabolism, fatty acid synthesis, and glycolysis. Proteins were curated only when they were quantified across all 9 cell lines and presented as heatmaps. Comparison of approximately 90 metabolic enzymes revealed two clear patterns. First, enzymes in the TCA cycle were mostly upregulated, with some cell-type-specific variations (Figs. 2*C* and S5*A*). Second, several enzymes involved in 1-carbon metabolism and fatty acid synthesis pathways showed significant upregulation or downregulation observed across all the tested cell lines (Fig. 2*D*). For example, thymidylate synthase (TYMS) and dihydrofolate reductase (DHFR), which catalyze the synthesis of dihydrofolate and tetrahydrofolate, respectively, showed strong downregulation (Figs. 2, *D* and *E*, and S5*B*). A previous study reported that sensitivity to TYMS and DHFR inhibition in blood cells by methotrexate (MTX) varies significantly, mainly because of thymidine supplementation through the serum (13). Our data show a significant downregulation (∼4-fold) in TYMS and DHFR protein levels after HPLM culture compared with DMEM culture in multiple cell lines examined here. Therefore, it will be interesting to test how much of the change in drug sensitivity is due to thymidine metabolite supplementation through the media *versus* overall changes in the target metabolic enzyme (13). Overall, our in-depth proteomic analysis allows for comprehensive identification of common features in proteome changes. This may also enable researchers to explore how variations in protein levels impact sensitivity or resistance to stress or pharmacologic interventions of metabolic enzymes.

When the proteins specifically localized to lysosomes were curated, the median change for 55 proteins was less than 25% across all 9 cell lines (Fig. 2*F*). This contrasts with earlier reports that lysosomal mass decreased several-fold in HeLa cells based on flow cytometry analyses upon LysoTracker treatment (23). This discrepancy may stem from the limitations of using LysoTracker intensity to measure lysosome mass, since LysoTracker intensity is affected by the acidity of endolysosomal lumens, cellular uptake of the dye, and background staining of cellular membranes, which is a known limitation of this dye. The mitochondrial proteome showed an overall increase across the cell lines, albeit a small amount (Fig. 2*G*), with HCT116 cells exhibiting an average increase of 63% (log_2_ 0.71) across 929 proteins. This is accompanied by nearly a two-fold increase in the mass of mitochondrial ribosomal proteins in HCT116 cells, potentially indicating increased mitochondrial biogenesis (Supplemental Fig. S5*C*). Consistent with the presence of selenium in HPLM but not in DMEM, selenoproteins GPX1, GPX4, and TXNRD1 were upregulated across most cell lines, along with the cystine transporter SLC7A11, confirming at the proteome level the ferroptosis-protective role of physiological media reported previously (19).

Lastly, an intriguing pattern was observed in the regulation of ribosomal proteins (r-proteins) across 9 cell lines (Fig. 2*H*). HEK293T cells showed nearly a 50% reduction in r-proteins, while other cell lines experienced smaller changes of less than 25%, if any.

Interestingly, the stoichiometry of 60S and 40S subunits also exhibited heterogeneous patterning, with HCT116 cells showing a reduction in 40S, while DU145, PANC1, and PC3 cells showed a reduction in 60S (Fig. 2*I*). Notably RIOK3 and RNF10, factors involved in initiation ribosome quality control (iRQC) (48, 49, 50) are significantly decreased (2-fold) in HEK293T cells, while both genes are notably upregulated in the other cell lines, suggesting altered iRQC in HPLM-cultured HEK293T (Supplemental Fig. S5*D*). Together, the systematic proteome data provide a quantitative overview of how the organellar proteome is reshaped across different cell types.

### HPLM Causes Cell-Type-Specific Reshaping of Mitochondria

**Fig. 3:**
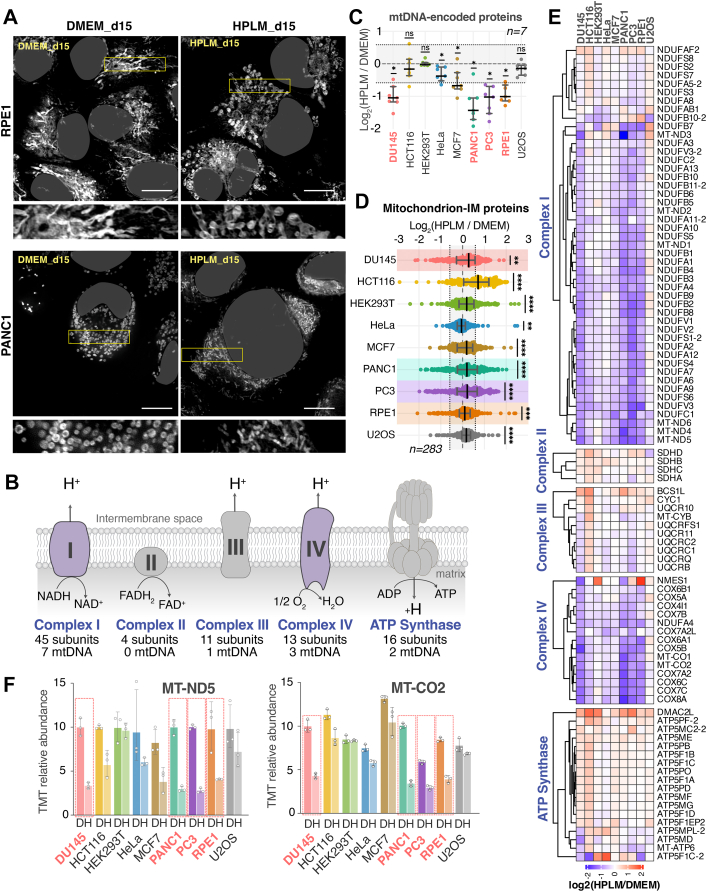
**HPLM causes cell-line-specific alterations in mitochondrial morphology and protein composition**. *A*, high-resolution imaging of mitochondria in live RPE1 and PANC1 cells treated with DMEM or HPLM for an extended period using MitoTracker Deep *Red*. Scale bar: 10 μm. *B*, schematic of the electron transport chain (ETC). mtDNA-encoded proteins are part of most ETC complexes except for complex II. *C*, dot plot of curated mtDNA-encoded proteins quantified in all 3 MS runs across the nine cell lines measured by TMT-proteomics. Mean ± S.E.M. for three biological replicates. The *grey shaded* area between the *dotted lines* indicates a ±1.5 ratio change. Cell lines with an average reduction of over 2-fold in MT proteins are highlighted in red. *p*-values were calculated using a one-sample Wilcoxon signed-rank test for a theoretical median of 0 to determine whether the differences between the two culture media were statistically significant (α = 0.01). The *grey shaded* area between the *dotted lines* indicates a ±1.5 ratio change. *D*, the scatter plot displays the Log_2_ ratio of HPLM/DMEM for mitochondrial inner-membrane proteins (MitoCarta3.0 annotation) across the nine cell lines. *Thick black lines* represent the median ratio values (± interquartile range) of 283 representative proteins (n = 3 biological triplicate experiments). *p*-values were calculated the same as in part C. *E*, heatmap representation of the abundance difference for ETC components curated from the proteomics dataset. Each data point represents the mean of three biological replicates. *F*, bar graphs for MT-ND5 (*left*, complex I) and MT-CO2 (*right*, complex IV) TMT-signal normalized to the DU145 DMEM condition. Mean ± S.D. for three biological replicates.

We examined the effect of HPLM on mitochondrial morphology using MitoTracker Deep Red, a fluorescent probe that covalently binds to mitochondrial proteins, allowing it to stain mitochondria largely independently of membrane potential (Supplemental Fig. S6*A*). Mitochondria in DMEM-grown HCT116 and PANC1 are small, globular structures, whereas those in U2OS, RPE1, or PC3 cells displayed elongated, tubular shapes (Figs. 3*A* and S6*B*). HCT116 and PANC1 cells harbor a KRAS mutation, which is associated with fragmented mitochondrial morphology. After culturing the cells in HPLM for either five or 15 days, we observed no noticeable difference in the mitochondria morphology in HCT116, U2OS, and PC3 cells. In contrast, RPE1 and PANC1 cells exhibited dramatically changed mitochondrial morphology, but with opposite patterns of fusion and fission. Mitochondria in RPE1 cells were notably swollen and fragmented after culturing them in HPLM, whereas those in DMEM-cultured cells displayed a typical tubular structure (Figs. 3*A*, S6*C*). PANC1, on the other hand, exhibited fragmented mitochondria in DMEM but showed tubular networks in HPLM culture (Fig. 3*A*). Phosphorylation status of DRP1 (DNM1L) at Serine 616 decreased slightly across the nine cell lines, which did not correlate with the heterogeneous changes in mitochondrial morphology (Supplemental Fig. S6*D*). Overall, HPLM culture caused varying degrees of changes in mitochondrial morphology across different cell lines, in a bi-directional fusion-fission rebalance that did not correlate with pS616 DRP1 status. Mitochondrial morphology reflects the balance between continuous fusion and fission cycles (51, 52, 53), and HPLM shifted this balance in a bi-directional manner across cell types.

Mitochondria are central to cellular metabolism, responsible for ATP production, redox balance, and biosynthesis of essential metabolites (54). Since mitochondria tend to fuse when cells rely on oxidative phosphorylation and fragment when OXPHOS dependence decreases (55), we next examined the effect of HPLM on OXPHOS. OXPHOS is governed by the electron transport chain (ETC) complexes located on the inner mitochondrial membrane. The ETC comprises four distinct complexes that generate proton gradients, which are then used by ATP synthase to produce ATP (Fig. 3*B*). Essential for forming ETC complexes are mitochondrial DNA (mtDNA)-encoded proteins. Mitochondria produce ∼13 proteins from mtDNA using their own ribosomes, while the rest of the roughly 1100 mitochondria-located proteins are imported from the cytosol. Upon HPLM culture, the proteins encoded by mtDNA showed a significant decrease in DU145, PANC1, PC3, and RPE1 cells (Fig. 3*C*). Since the ETC is located in the mitochondrial inner membrane, we compared the abundance of 71 inner membrane resident proteins (Fig. 3*D*). We found that while most proteins remained unchanged, a subset of proteins, primarily representing ETC components, showed a pronounced decrease in DU145, PANC1, PC3, and RPE1 cells. When 94 of the ETC proteins were curated and plotted, it became clear that complex I and complex IV proteins in DU145, PANC1, PC3, and RPE1 cells are consistently decreased when cultured in HPLM compared to DMEM, while the other complexes remain mostly unchanged (Fig. 3*E*). In particular, MT-ND5 in Complex I and MT-CO2 in Complex IV exhibited the strong downregulation (Fig. 3*F*). A previous study reported increased mitochondrial respiration in Plasmax-cultured HeLa, A549, and Huh7.5 cell lines based on Seahorse analysis (23). These differences could be due to the heterogeneous response of the cell lines, as the U2OS cell line showed a slight increase in ETC abundance in our study.

### Phosphoproteome Remodeling Highlights altered Mitophagy and mTORC1 Signaling

**Fig. 4:**
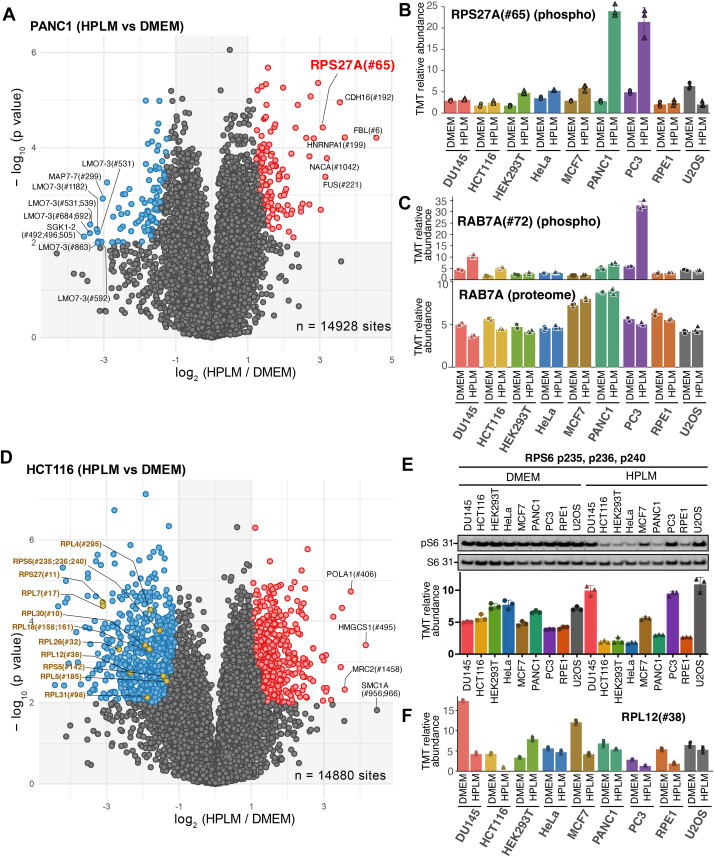
**Phosphoproteome remodeling highlights altered mitophagy and mTORC1 signaling**. *A*, analysis of the phosphoproteomic data is presented as a volcano plot of the -log10-transformed *p*-value *versus* the log_2_-transformed ratio of HPLM/DMEM conditions for PANC1 cells. Individual value represents the peptide containing the phosphorylation site. *p*-values were calculated using a two-sided Welch’s *t* test (adjusted to 1% FDR for multiple comparisons, S0 = 1). Among the statistically significant hits, sites that are significantly upregulated are circled in red, while those that are downregulated are in *blue*. A total of 14,928 sites were quantified without missing values. n = 3 biological replicates. *B*, scaled value of phospho-Serine at 65 of ubiquitin is shown as bar graphs. Mean ± S.D. for three biological replicates. PANC1 and PC3 show over 8- and 4-fold increase, respectively, whereas U2OS shows a decrease. *C*, scaled values of phospho-Serine72 of Rab7 (*top*) and the Rab7 total protein level (*bottom*) are shown as bar graphs. Mean ± S.D. for three biological replicates. *D*, HCT116 phospho-enriched peptide quantification is shown as -log10-transformed *p*-value *versus* the log2-transformed ratio of HPLM/DMEM conditions. Individual value represents the peptide containing the phosphorylation site. *p*-values were calculated using a two-sided Welch’s *t* test (adjusted to 1% FDR for multiple comparisons, S0 = 1). Phosphosites that are significantly upregulated are circled in *red*, while those that are downregulated are in *blue*. A total of 14,880 sites were quantified without missing values. n = 3 biological replicates. *E*, extracts from 9 cell lines cultured in either DMEM or HPLM for 2 weeks were probed with anti-pS235,236 and total S6 antibodies (*top*), and the corresponding phosphoproteomics data are shown below. The bar graph shows mean ± SD for three biological replicates. *F*, scaled values of phospho-Serine38 of RPL12 are plotted as a bar graph. Mean ± S.D. for three biological replicates.

Given the broad impact of HPLM culture on the stability of total and organellar proteomes, we curated our phosphoproteomics dataset, collected simultaneously, to identify signaling pathway responses that may explain biological differences (Supplemental Fig. S7). The phosphorylation event that is relevant to mitochondrial quality control (mitoQC) processes caught our attention first, since the dramatic loss of ETC complexes observed in the proteomic data could affect mitochondrial membrane potential and trigger mitoQC (Fig. 4*A*). Notably, Ub Ser65 (RPS27A #65), a marker of PINK1-dependent mitophagy (56, 57), was one of the most significant hits identified in PANC1 cells, showing an 8.5-fold increase after HPLM culture (Fig. 4, *A* and *B*). A 4.3-fold increase in phosphorylation of Ub S65 was also observed in PC3 cells (Fig. 4*B*). Interestingly, PC3 cells also upregulate RAB7A S72, a target of TBK1 kinase that promotes mitophagy (Fig. 4*C*) (58, 59, 60, 61). To better measure mitophagy flux during HPLM culture, we used a mito-Keima reporter system (46), a dual-excitation fluorescent protein (62) that allows ratiometric analysis of mitochondrial degradation in lysosomes (Supplemental Fig. S7*B*). We specifically engineered PANC1 and PC3 cells, which showed a significant increase in pS65 levels during HPLM culture. Cells stably expressing the mito-Keima reporter were FACS-sorted for equal expression, then cultured in DMEM or HPLM for 15 days, and subsequently analyzed by flow cytometry. As a positive control, we added mitochondrial depolarizing agents, antimycin and oligomycin (AO), for 6 h. Upon AO treatment, the Red/Green Keima ratio increased significantly, indicating that the reporter system is working (Supplemental Fig. S7*C*). However, when comparing the Keima ratio of the DMEM and HPLM cultured cells, there were no dramatic changes at the whole sample level. Examining individual cells’ fluorescence signal changes showed a slight increase in the gated population during HPLM culture, especially in PANC1 cells, suggesting that mitophagy might have been triggered in a small number of cells, which could have contributed to the pS65-Ub signal, given the low initial level in the DMEM (Supplemental Fig. S7*D*). Overall, the data indicated that some cell lines may exhibit increased steady-state mitophagy signaling during HPLM culture. However, a more in-depth analysis is needed for this minor population undergoing mitophagy.

Another notable aspect of the phospho-signaling change, particularly in HCT116 cells cultured in HPLM, was the reduction in phosphorylation of ribosomal proteins (Fig. 4*D*). Several sites on both RPL and RPS proteins were significantly decreased, including RPS6 S235, S236, and S240, suggesting a reduced mTORC1 signaling. Other cell lines, such as HeLa and HEK293T, also showed a dramatic decrease in pS6 signal, except for the PC3 and U2OS cell lines, which were confirmed by immunoblotting results (Fig. 4*E*). RPL12 S38 was also one of the sites significantly downregulated upon HPLM culture (Fig. 4*F*). This site is known to be phosphorylated by CDK1 and/or CDK2 during mitosis, which is suggested to regulate the translation of mRNA related to mitosis (63). Therefore, the decrease in RPL12 pS38 suggests a reduction in mitosis and cell division.

### Reduced Cell Cycle Activity is a Common Feature of Cells Cultured in HPLM

**Fig. 5:**
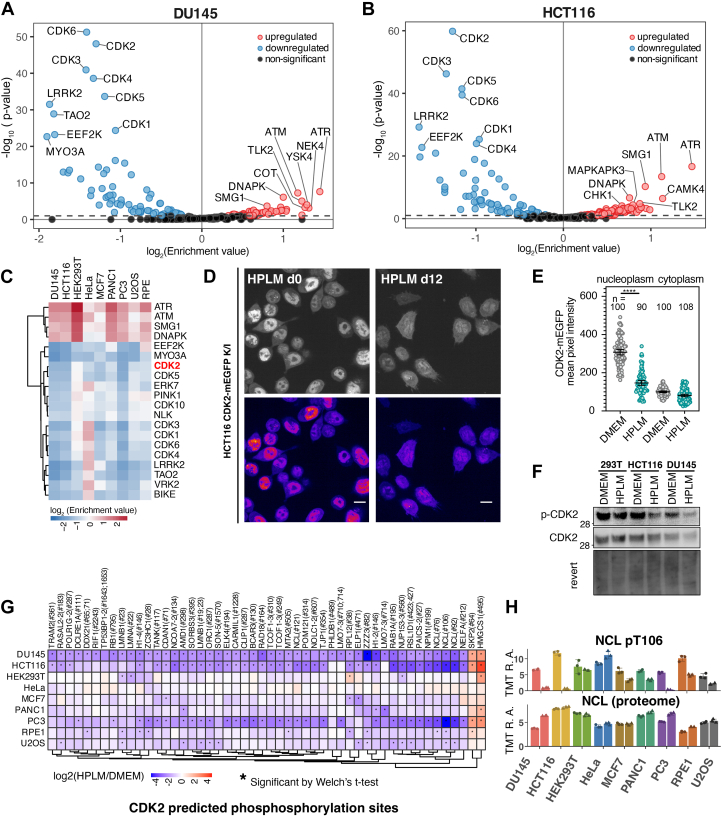
**Reduced cell cycle activity is a general feature of cells cultured in HPLM**. *A* and *B*, motif enrichment analysis to identify kinase-specific signatures from phosphoproteomics data of DU145 (panel *A*) and HCT116 (panel *B*) shows a significant and consistent downregulation of CDK activity and upregulation of ATM/ATR activities. *C*, heatmap displaying the Log_2_ enrichment of activity in HPLM culture for the top 20 kinases, curated from the motif enrichment analysis. *D*, live imaging analysis of HCT116 cells endogenously expressing CDK2-mEGFP to study CDK2 subcellular localization. Scale bar: 10 μm. *E*, the mean pixel intensity of nuclear and cytoplasmic areas in individual cells in panel D was quantified and plotted with mean ± S.E.M. n = 100, 90, 100, 108 cells quantified. Significance was calculated using a two-tailed unpaired *t* test with Welch’s correction (∗∗∗∗, *p*< 0.0001). *F*, Wild-type HEK293T, HCT116, and DU145 cell lysates were gathered after 15 days of culture in DMEM or HPLM and resolved using the indicated antibodies. *G*, heatmap showing phosphopeptides predicted as CDK2 substrate sites, curated from the phosphoproteomics dataset. Each point indicates the mean of three biological replicates. ∗denotes statistically significant hits. *H*, scaled values of the NCL pT106 (*top*) and the total NCL protein levels (*bottom*) are plotted as a bar graph. Mean ± S.D. for three biological replicates. RA, relative abundance.

To postulate changes in kinase activities upon HPLM culture, the Kinase Library database was utilized for kinase-substrate predictions across the nine cell lines, using >10,000 single, localized phosphosites quantified in all three mass spectrometry runs (44). Most notably, five of the cell lines (DU145, HCT116, MCF7, PANC1, and PC3) exhibited predicted decreases in CDK1-6 activity based on reduced phosphorylation of their predicted substrates, along with increased activity of ATM and ATR (Figs. 5, *A* and *B*, and S8*A*). ATM and ATR sense replication stress or DNA damage and deactivate CDK2, thereby acting as regulators of cell cycle checkpoints (64, 65). Additionally, key downstream regulators of cell division, such as CDC20 and CDC25 (Supplemental Table S1), also showed a consistent decrease across all HPLM-cultured cell lines, except for HEK293T and HeLa cells. Collectively, cell cycle arrest emerged as the main signaling response to HPLM culture, consistently seen across several cell lines.

To better understand how HPLM affects CDKs, we engineered HCT116 cells to endogenously express CDK2-mEGFP and observed the subcellular localization of CDK2 (Supplemental Fig. S8*B*). Live imaging of HCT116 CDK2-mEGFP knock-in (K/I) cells showed that CDK2 is located in both the nucleus and cytoplasm, but it is more concentrated in the nucleus when cultured in DMEM (Supplemental Fig. S8*C*). After 5 days in HPLM media, there was a subtle but noticeable increase in nucleoplasm-localized CDK2-mEGFP, as indicated by the average mean pixel intensity (Supplemental Fig. S8, *C* and *D*). However, by day 12, GFP intensity significantly declined, and the distribution between the nuclear and cytoplasmic regions changed, with a clear reduction in nuclear CDK2-mEGFP signal (Fig. 5, *D* and *E*). This result aligns with the observed reduction in phosphorylation levels of predicted CDK2 substrates. To assess CDK2 activation status directly, we performed Western blot analysis using an antibody specific for the activating phosphorylation site p-Thr160 (Fig. 5*F*). CDK2 p-T160 levels are dramatically reduced in HCT116 and DU145 cells grown in HPLM for 15 days, suggesting impaired cell cycle progression. HEK293T cells showed a mild reduction in p-CDK2 signal, which matched the overall kinase-substrate prediction (Supplemental Fig. S8*A*).

The prediction for CDK2 inactivation in HPLM is based on shared sequence motifs of de-enriched phosphosites. To validate the quality of this prediction, we analyzed the top contributing sites for known CDK2 substrates (Fig. 5*G*). Among the top hits are nucleolin (NCL) and nucleophosmin (NPM1) (Fig. 5*H*). Significant dephosphorylation of NCL T106 and NPM1 T199 occurred in the DU145, HCT116, and PC3 cell lines in HPLM culture. NPM1 is a nucleolar phosphoprotein important for ribosome biogenesis, but also has a function in the centrosome, where it is targeted by CDK2/cyclin-E (66). Previous work to identify CDK2 substrates used a bump-and-hole approach to engineer a CDK2 capable of utilizing ATP analogs to thiophosphorylate peptides for mass spectrometry analysis. This strategy identified NPM1 and NCL phosphorylation by CDK2, as well as RPL12 S38 phosphorylation (67). Overall, our data indicate that the HPLM activates cell cycle checkpoint responses and slows down cell cycle progression. Consistently, the growth of all 9 cell lines over 15 days in HPLM culture decreased compared to DMEM-cultured cells, although HPLM notably affected specific cells such as HEK293T and HCT116 (Supplemental Fig. S9*A*). The overall decline in cell proliferation during HPLM culture aligns with our observation of decreased CDK2 activity.

### HPLM Reduces Enzymes in the Pyrimidine Synthesis and the Mevalonate Pathway

With the HPLM-induced changes to mitochondrial composition and the enzymes involved in ATP-generating pathways, we hypothesized that culture in physiological media could have comprehensive effects on some key metabolic processes. Additionally, our previous studies have established that the protein half-lives of rate-limiting enzymes involved in pyrimidine and mevalonate pathways are regulated by mTORC1 activity (24, 68); therefore, the reduction of mTOR activity in HPLM that we observed could also influence their endogenous expression.

**Fig. 6:**
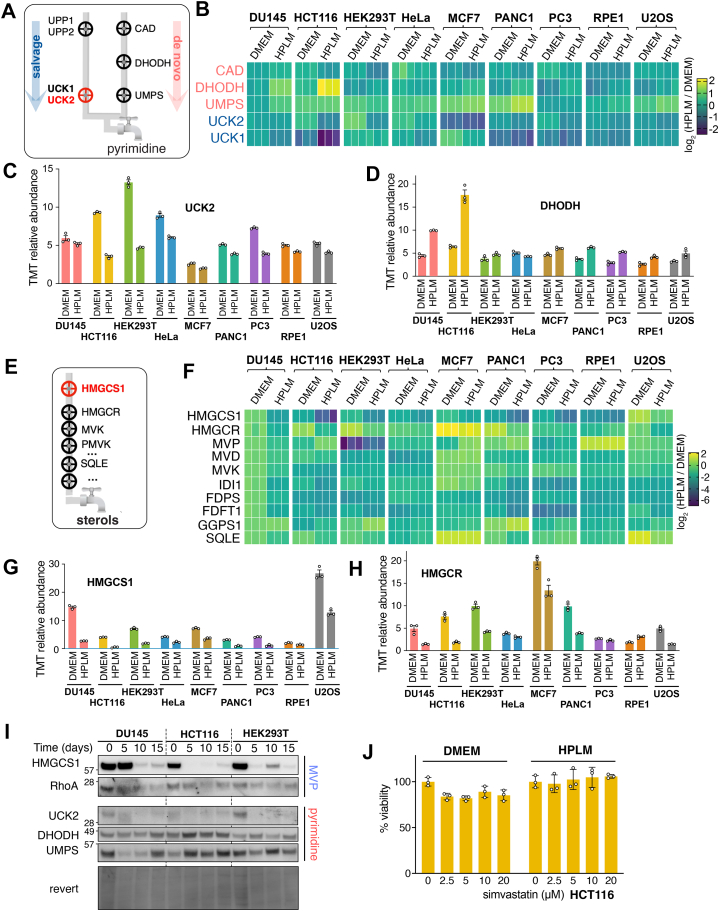
**HPLM decreases the expression of enzymes in pyrimidine synthesis and the mevalonate pathway**. *A*, a schematic illustrating two pyrimidine biogenesis pathways, salvage and *de novo*, along with the enzymes involved in each pathway. *B*, heatmap showing the protein levels, normalized to the DU145 DMEM condition, of pyrimidine metabolic enzyme abundance. *C* and *D*, relative abundance (TMT-scaled value) of UCK2 and DHODH is shown as bar graphs. Mean ± SD for three biological replicates. *E*, a schematic illustrating the sterol biogenesis pathway. *F*, heatmap displaying the relative abundance of sterol metabolic enzymes normalized to the DU145 DMEM condition. *G* and *H*, relative abundance (TMT-scaled value) of the two initial enzymes in the mevalonate pathway, HMGCS1 and HMGCR, shown as bar graphs. Mean ± S.D. for three biological replicates. *I*, immunoblotting assay with indicated antibodies for metabolic enzymes in sterol and pyrimidine synthetic pathways confirms the proteomic data. *J*, the viability of HCT116 cells cultured in DMEM or HPLM for 15 days was tested after increasing concentrations of Simvastatin treatment, using nuclear staining and flow cytometry-based analysis.

Pyrimidine biogenesis is regulated by two processes: salvage and *de novo* (Fig. 6*A*). Comparison of five enzymes in these pathways revealed distinct differences among the cell lines (Fig. 6*B*). In particular, the rate-limiting enzyme of the pyrimidine salvage pathway, UCK2, was downregulated across 9 cell lines (Fig. 6, *B* and *C*). This is consistent with the reduced mTORC1 activity in HPLM culture and our previous finding that mTORC1 deactivation triggers selective UCK2 degradation via the CTLH-WDR26 E3 ubiquitin ligase complex (68). We also demonstrated that when UCK2 levels decrease, *de novo* pyrimidine synthesis increases as a compensatory mechanism (68). This was reflected in the proteomics data, as DHODH, a key enzyme in the de novo pathway, exhibited an overall increase in its expression (Fig. 6*D*). However, it is likely that cellular requirements for pyrimidine biogenesis are lower in HPLM-cultured cells because the overall cell cycle and DNA replication processes have slowed down. Human serum uridine levels fluctuate between 3 to 9 μM, which can affect the balance between de novo synthesis and salvage pathways (69). Notably, physiological media contains 3 μM of uridine, whereas DMEM does not. The use of dialyzed serum completely depletes the DMEM of exogenous uridine supplementation. Therefore, the overall changes in the pyrimidine synthetic pathway enzymes may have been caused by multiple factors, including uridine supplementation, uric acid level in the media, changes in mTORC1 activity, and demand on the DNA replication, thereby influencing the synthesis of pyrimidine derivatives.

Another essential metabolite in cells is mevalonate, an essential precursor for sterols and isoprenoids (Fig. 6*E*). From a therapeutic standpoint, statins have been developed to lower blood cholesterol levels by inhibiting the mevalonate pathway enzyme HMGCR. Results of repurposing statins as anti-cancer agents have been largely inconsistent, partly due to feedback inhibition that increases HMGCR levels by over 50-fold, thus impacting the intended downregulation of its activity. Our prior work has linked mTORC1 deactivation to the selective degradation of HMGCS1, the initial enzyme in the mevalonate pathway (24). As with the pyrimidine pathway enzymes, the 9 cell lines displayed heterogeneity in their expression of sterol biosynthesis enzymes (Fig. 6*F*). However, the levels of HMGCS1 and HMGCR were significantly downregulated in most cell lines (Fig. 6, *G* and *H*).

Immunoblotting for pyrimidine and mevalonate pathway enzymes was consistent with the proteomic data (Fig. 6*I*). In DU145, HCT116, and HEK293T cells, UCK2 was lowly expressed in HPLM culture, with the highest levels at day 0. Meanwhile, DHODH expression increased in HCT116 and DU145 cell lines after 15 days in HPLM culture. UMPS levels appeared to fluctuate during the time course experiment, suggesting cell-specific metabolic adaptation over the 2 weeks in physiological media. Similar to UCK2, HMGCS1 was most highly expressed at day 0 and substantially reduced by day 15. Although we previously observed that acute depletion of HMGCS1 upregulates RhoA expression as a feedback response, we did not observe this phenotype after long-term culture in physiological media (70).

Compounds targeting the mevalonate pathway have been clinically in use for several decades, as exemplified by the statin family, which inhibit HMGCR. We investigated the effect of culture media on the viability of HCT116 cells following simvastatin treatment. Our prior study demonstrated that, among many cell lines tested, HCT116 cells are particularly resistant to simvastatin when cultured in DMEM, and we wondered if HPLM culture has any impact on this due to the dramatic loss of HMGCS1 and HMGCR, the first two enzymes in the mevalonate pathway (70). After 10 days of HPLM culture, HCT116 cells did not demonstrate increased sensitivity to statin compared to cells maintained in DMEM (Fig. 6*J*). A previous drug screening study calculated the differential sensitivity across three blood cell lines (NOMO1, P12-Ichikawa, and SEM) based on culture media and found that the influence of the media on cell responses to several statin compounds was not uniform (13). However, cells grown in HPLM *versus* regular culture media supplemented with dialyzed FBS responded to simvastatin with an average differential sensitivity score of −0.208, indicating that the culture media did not drastically alter response, similar to our result.

## Discussion

Our comprehensive proteomic comparison of 9 cell lines cultured in DMEM *versus* HPLM reveals that physiological nutrient conditions trigger widespread, cell-type-specific proteome remodeling. These changes affect mitochondrial metabolism, CDK and mTORC1 signaling, and ribosome homeostasis. These findings demonstrate that conventional culture media mask substantial metabolic and signaling states that may better represent *in vivo* conditions, with direct implications for drug sensitivity testing. Over the past decade, physiological media such as HPLM and Plasmax have been developed to better mimic the nutrient environment of human plasma (17, 19). While studies using these media have begun to uncover previously overlooked metabolic wiring and metabolite-protein interactions, our understanding of how the broader metabolic proteome is reprogrammed under physiological conditions has remained limited. A prior study assessed proteome changes in MDA-MB-231BR and CRL-1620 cells upon Plasmax treatment, but only ∼1600 proteins were quantified without missing values, covering less than 14% of the expressed proteome (71). Our study addresses this gap with deep proteomic coverage of over 9000 proteins across 9 cell lines, providing a systematic view of the proteomic consequences of physiological culture.

We conducted a comprehensive comparison of the proteomes of cells grown in standard medium (DMEM) and physiological medium (HPLM) across nine different cell lines and focused on changes in subcellular organelle-specific proteomes and metabolic enzymes. We cultured cells in HPLM for approximately 15 days (over three passages) to ensure near-complete proteome replacement, given variable doubling times across cell lines ranging from 16 to 35 h. This duration was based on protein turnover kinetics, where 5 to 6 divisions are required to replace more than 98% of the original protein pool.

Dramatic adaptation in mitochondrial morphology and metabolic processes were observed, but these changes were surprisingly cell-type-specific. PANC1 cells showed fragmented mitochondrial morphology in DMEM, but highly fused mitochondria were observed when grown in HPLM. Conversely, RPE1 cells exhibited a tubular mitochondrial network in DMEM but showed highly fragmented mitochondria in HPLM. The observed changes in mitochondrial morphology appear to result from multiple factors, including activation of mitophagy (nutrient limitation can also induce mitochondrial fusion as a protective mechanism (72)) and alterations in OXPHOS, but are not related to DRP1 phosphorylation status via MAPK signaling. Apart from morphological differences, most cell lines showed significantly lower protein levels of the electron transport complexes I and IV, suggesting a downregulation of OXPHOS. Enzymes involved in the TCA cycle were also consistently downregulated across cell types.

Mitochondrial function requires the import of over 1000 proteins synthesized by cytosolic ribosomes using various import machineries based on the presence of mitochondrial presequences and other targeting motifs. ETC complex assembly poses a unique challenge for cells to balance the stoichiometry of nuclear-encoded and mitochondria-encoded subunits. We observed strong downregulation of the seven mtDNA products detected by proteomics, as well as many of the cytosolically translated components of complex I and complex IV in response to HPLM culture. Since all four electron transport chain (ETC) complexes require cytosolic import of their subunits for assembly, yet only complexes I and IV were decreased, a post-translational degradation mechanism may be responsible for the selective complex downregulation. Within the inner mitochondrial membrane (IMM), protein quality control is carried out by two major mitochondrial proteases, i-AAA (YME1L) and m-AAA (SPG7 and AFG3L2), which degrade damaged, misfolded, or imbalanced proteins (73). Within the mitochondrial matrix, CLPP, a serine quality control protease, has been shown to degrade the NADH dehydrogenase module of complex I in response to the ROS inducers rotenone and antimycin A (74). Since HPLM culture resulted in downregulation of complex I and complex IV, it is possible that protease activity is responsible for selective ETC complex quality control. Notably, U2OS cells did not exhibit changes in ETC or TCA enzymes, underscoring their distinct response to alterations in the metabolic environment. Together, various mitochondrial processes were altered collectively under HPLM culture, but each process was specific to the cell type. Since many studies using physiological media focus on mitochondrial metabolism, it will be important to test different cell lines to assess the targeted metabolic changes.

Our data also revealed a disconnect between morphological changes and proteome remodeling. DU145 cells exhibited almost no visible morphological alteration upon HPLM culture, yet 16% of their proteome was significantly changed. Conversely, MCF7 cells displayed notable morphological differences after extended HPLM culture but showed only 4.8% significant proteome changes. This disconnect suggests that morphological adaptations and proteome remodeling operate through partially independent mechanisms. Cytoskeletal reorganization and membrane remodeling, for instance, can occur without substantial changes in protein abundance, while metabolic adaptations may not immediately manifest as visible structural changes. The implication for researchers using physiological media is that morphological assessment alone is insufficient to predict the extent of underlying molecular changes. Cell lines that appear morphologically stable may nonetheless harbor substantial metabolic and signaling alterations that could affect experimental outcomes, including drug responses.

The reduced CDK activity we observed across multiple cell lines likely contributes directly to the proteome remodeling induced by HPLM. CDK2 requires nuclear localization through cyclin binding to execute its functions in cell cycle regulation and DNA replication (75), and the reduced nuclear CDK2-mEGFP signal we observed in HPLM-cultured cells indicates impaired CDK2 activation. Full CDK2 activity also requires T-loop phosphorylation at Thr160 by CDK-activating kinase (CAK) (76, 77, 78, 79, 80), and our Western blot data confirmed marked reductions in p-T160 levels. This CDK inactivation has broad implications for proteome composition. CDK substrates include not only cell cycle regulators but also ribosome biogenesis factors such as nucleolin and nucleophosmin (66, 67), and the dephosphorylation of these substrates likely contributes to the observed changes in ribosomal protein levels. The connection between CDK activity and ribosome homeostasis is particularly relevant given the heterogeneous ribosomal protein changes we observed, including the nearly 50% reduction in HEK293T cells. Ribosome levels are normally tightly regulated at multiple stages to maintain constant ribosome density (81, 82, 83), and CDK-dependent phosphorylation of ribosome biogenesis factors may be essential for this homeostasis. The downregulation of RIOK3 and RNF10, factors that mediate ribosome quality control in response to subunit imbalance (48, 49, 50), specifically in HEK293T cells, suggests that different cell lines employ distinct mechanisms to cope with the metabolic stress imposed by physiological media.

Our data support a model in which specific metabolites in HPLM trigger coordinated changes in nutrient-sensing pathways that rewire cellular signaling and reshape the proteome. An additional layer of rewiring involves the methionine-SAM axis. MAT2A and MAT1A, the only two proteins significantly regulated across all 9 cell lines, catalyze SAM biosynthesis from methionine, and their strong induction likely compensates for the approximately 6-fold lower methionine concentration in HPLM. In parallel, SAM-dependent methyltransferases spanning histones (DOT1L, EZH2, SMYD2, and SETD6), tRNA (METTL2B), rRNA (METTL5), and protein N-termini (NTMT1) were coordinately depleted while arginine methyltransferases remained unaffected, consistent with a model in which physiological methionine levels limit SAM availability and prompt cells to reduce their methyltransferase inventory (84). HPLM contains metabolites at physiological concentrations that differ substantially from conventional media, including elevated uric acid, the presence of uridine (3 μM), and altered amino acid ratios (17). These metabolites directly engage nutrient-sensing pathways: reduced amino acid availability and altered ratios suppress mTORC1 activity, as evidenced by decreased phospho-S6K and phospho-S6 levels across all 9 cell lines. mTORC1 suppression has cascading effects on protein synthesis, autophagy, and the stability of specific metabolic enzymes. We previously demonstrated that mTORC1 deactivation triggers selective degradation of UCK2 via the CTLH-WDR26 E3 ligase complex (68) and HMGCS1 (24), consistent with the downregulation of these enzymes observed here. The convergence of reduced mTORC1 signaling and CDK inactivation creates a cellular state characterized by slower proliferation, reduced biosynthetic demand, and altered metabolic flux through pyrimidine and mevalonate pathways.

These proteome-level changes have direct implications for drug sensitivity that extend beyond simple metabolite-enzyme inhibition. Uric acid levels in HPLM are 10 times higher than in traditional media, and this concentration has been reported to inhibit UMPS, reducing sensitivity to pyrimidine analog prodrugs such as 5-fluorouridine (13, 17). Our data show that the effects on pyrimidine metabolism go further: enzymes involved in pyrimidine salvage, particularly UCK1 and UCK2, which together with UMPS convert the prodrug 5-FU into its active form, were consistently downregulated in HPLM cultures. HPLM culture therefore causes notable changes in levels of metabolic enzymes across the pyrimidine synthesis pathway, which may affect sensitivity to many pyrimidine analog prodrugs. For CDK inhibitors currently in clinical use, the baseline reduction in CDK activity and altered CDK substrate phosphorylation in HPLM-cultured cells may affect therapeutic windows. Our finding that HCT116 cells did not show increased simvastatin sensitivity despite reduced HMGCS1 and HMGCR levels suggests that feedback mechanisms or alternative metabolic routes may compensate for enzyme downregulation, highlighting the complexity of predicting drug responses from proteome data alone.

Together, these findings underscore the importance of considering culture conditions when evaluating metabolic drug candidates. The signaling state and proteome composition of cells in physiological media may better reflect the *in vivo* tumor environment, and accounting for these differences will be essential for improving the translational relevance of preclinical drug testing.

### Limitations of the Study

Our proteomic analysis captures a single snapshot within an ongoing process of proteome remodeling rather than a definitive equilibrium state, as evidenced by the significant morphological changes some cell lines exhibited over the course of several passages. Follow-up analyses of various features, including CDK2 localization shifts and levels of metabolic enzymes, revealed notable differences between cells cultured in HPLM for 5 days *versus* 15 days. Protein levels fluctuated over time with distinct temporal patterns across proteins and cell lines, highlighting the difficulty of defining a single, universal adaptation timepoint. Because HPLM culture modulates key regulators such as mTORC1 signaling, CDK2 activity, and cell cycle progression, it is possible that a stable proteomic steady state is never fully achieved under these conditions. The 15-day time point therefore represents one phase of an ongoing adaptation process rather than an equilibrium state. Tracking proteome changes over time in future studies may help distinguish transient responses from stable adaptations and identify the molecular determinants of this temporal heterogeneity. Additionally, the organellar proteome, particularly ribosomal proteins, displayed variable changes across different cell lines, and the source of this heterogeneity remains unknown. Since ribosomal proteins are regulated at multiple levels, further research into ribosome quality control factors or biogenesis could reveal cell-type-specific regulators.

## Data and Code Availability

• All unique identifiers and web links for publicly available datasets are presented within the main text or the supplementary materials. Mass spectrometry data associated with Supplemental Tables S1–S2 have been deposited to the MassIVE repository and are publicly available as of the date of publication, with the dataset identifier MSV000101371. The main proteomics and phosphoproteomics datasets from this study are publicly accessible online (https://ordureau-lab.shinyapps.io/HPLM_Shiny_App/). Users can explore the datasets at both gene-specific and site-specific levels.
• This paper does not report any original code.
• Any additional information required to reanalyze the data reported in this paper is available from the lead contact upon request.

## Supplemental Data

This article contains supplemental data.

## Conflict of Interest

The authors declare that they do not have any conflicts of interest with the content of this article.
