# Supplementary material for: Proteomic Profiling Reveals How Physiological Media Reshape Cancer Cell Proteomes and Signaling Networks
Source: Mol Cell Proteomics. 2026 Apr 15;25(5):101569. doi: 10.1016/j.mcpro.2026.101569 (PMC13195775; doi:10.1016/j.mcpro.2026.101569)
Supplement: Supplemental Material 1 [file mmc1.pdf]

TITLES OF SUPPORTING DATA MATERIALS

Supplemental **Figure S1-9**. Relevant to Figures 1-5.

Figure S1. Changes in cellular morphology induced by HPLM. Relevant to Figure 1.

Figure S2. Systematic analysis of proteomic changes induced by HPLM. Relevant to Figure 1.

Figure S3. Gene Ontology analysis of proteome changes induced by HPLM. Relevant to Figure 1.

Figure S4. Volcano plots of nine cell line proteomics. Relevant to Figure 2.

Figure S5. Metabolic proteome response to HPLM. Relevant to Figure 2.

Figure S6. Effect of HPLM on mitochondria morphology. Relevant to Figure 3.

Figure S7. Phosphoproteome remodeling upon HPLM culture across cell lines. Relevant to Figure 4.

Figure S8. Kinase motif enrichment analysis reveals a decrease in CDKs activity as a common feature of HPLM-cultured cells. Relevant to Figure 5.

Figure S9. Cell proliferation assay for nine cell lines. Relevant to Figure 5.

Supplemental **Tables S1-2**. Relevant to Figures 1-6

Table S1. Spreadsheet containing the proteomic changes (protein level) induced by HPLM. Relevant to Figure 1 and S3.

Table S2. Spreadsheet containing the phosphoproteomic changes (phosphorylation site) induced by HPLM. Relevant to Figure 4 and S6.

Figure S1.

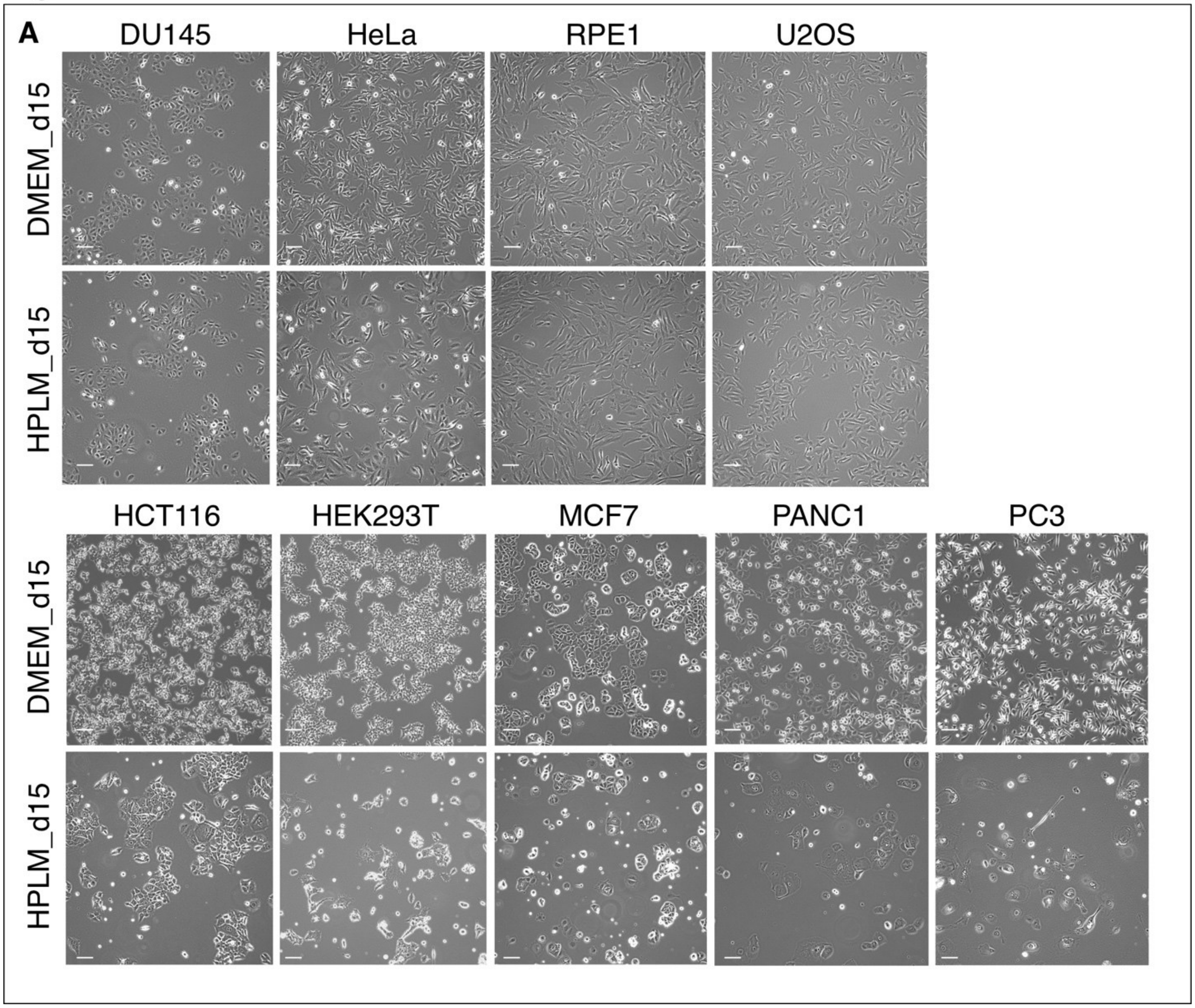

Figure S1. Changes in cellular morphology induced by HPLM (related to Fig.1)

(A) Morphology of cells grown in DMEM and HPLM was observed using inverted light microscopy. Scale bar = 100 µm. Top: cell lines with no significant morphological changes. Bottom: cell lines with notable morphological alterations.

Figure S2.

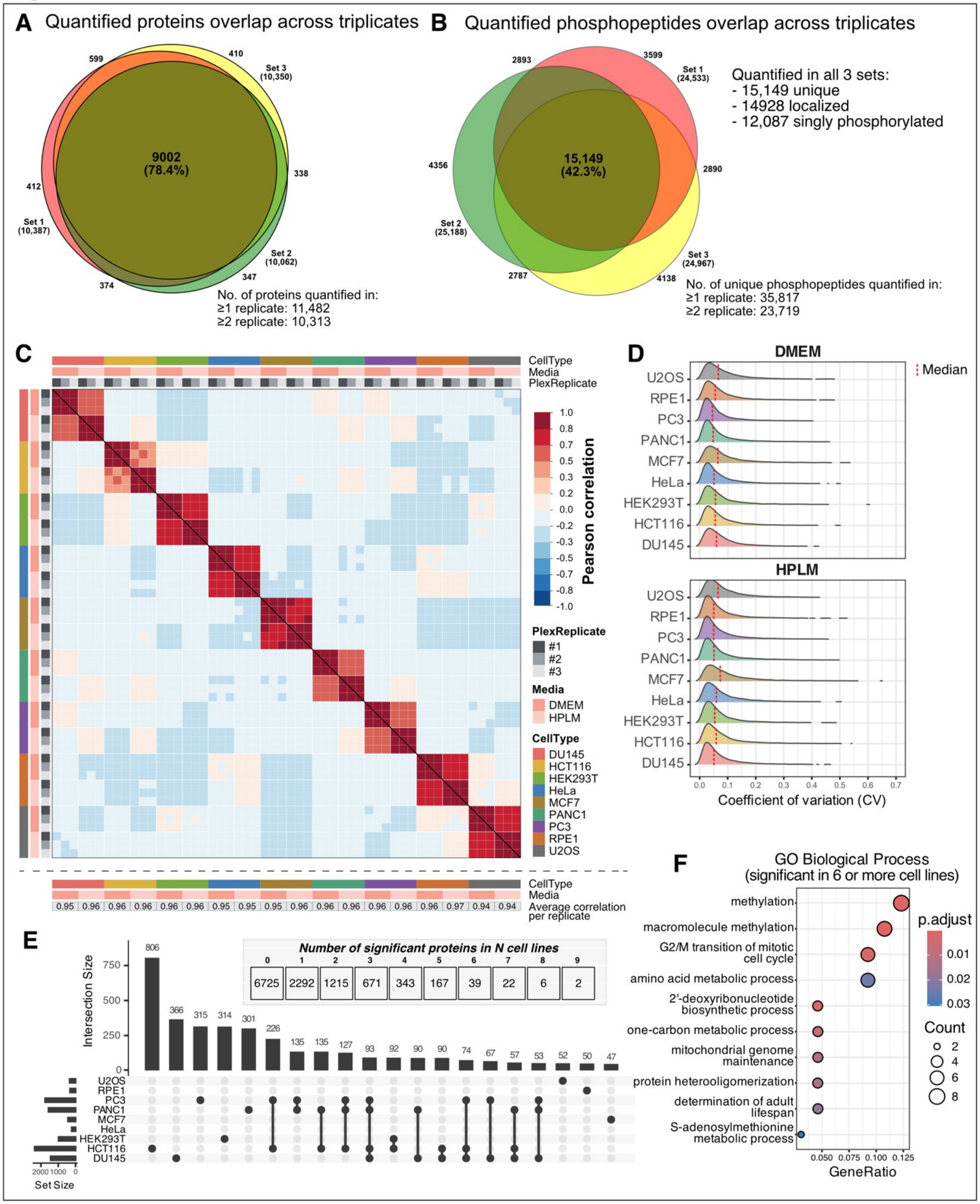
A Quantified proteins overlap across triplicates
599
410
Set 3 (10,350)
9002 (78.4%)
338
412
Set 1 (10,387)
Set 2 (10,062)
347
374
No. of proteins quantified in:
≥1 replicate: 11,482
≥2 replicate: 10,313
B Quantified phosphopeptides overlap across triplicates
2893
3599
Set 1 (24,533)
Quantified in all 3 sets:
- 15,149 unique
- 14928 localized
- 12,087 singly phosphorylated
4356
15,149 (42.3%)
2890
Set 2 (25,188)
Set 3 (24,967)
2787
4138
No. of unique phosphopeptides quantified in:
≥1 replicate: 35,817
≥2 replicate: 23,719
C
CellType
Media
PlexReplicate
Pearson correlation
1.0 0.8 0.7 0.5 0.3 0.2 0.0 -0.2 -0.3 -0.5 -0.7 -0.8 -1.0
PlexReplicate
#1
#2
#3
Media
DMEM
HPLM
CellType
DU145
HCT116
HEK293T
HeLa
MCF7
PANC1
PC3
RPE1
U2OS
CellType
Media
Average correlation per replicate
0.95 0.96 0.96 0.96 0.96 0.96 0.95 0.95 0.95 0.96 0.96 0.96 0.96 0.96 0.96 0.97 0.94 0.94
D
DMEM
Median
U2OS
RPE1
PC3
PANC1
MCF7
HeLa
HEK293T
HCT116
DU145
HPLM
U2OS
RPE1
PC3
PANC1
MCF7
HeLa
HEK293T
HCT116
DU145
0.0 0.1 0.2 0.3 0.4 0.5 0.6 0.7
Coefficient of variation (CV)
E
Intersection Size
750 500 250 0
806 366 315 314 301 226 135 135 127 93 92 90 90 74 67 57 53 52 50 47
Number of significant proteins in N cell lines
0: 6725
1: 2292
2: 1215
3: 671
4: 343
5: 167
6: 39
7: 22
8: 6
9: 2
U2OS
RPE1
PC3
PANC1
MCF7
HeLa
HEK293T
HCT116
DU145
2000 1000 0
Set Size
F
GO Biological Process
(significant in 6 or more cell lines)
methylation
macromolecule methylation
G2/M transition of mitotic cell cycle
amino acid metabolic process
2'-deoxyribonucleotide biosynthetic process
one-carbon metabolic process
mitochondrial genome maintenance
protein heterooligomerization
determination of adult lifespan
S-adenosylmethionine metabolic process
0.050 0.075 0.100 0.125
GeneRatio
p.adjust
0.01
0.02
0.03
Count
2
4
6
8

Figure S2. Systematic analysis of proteomic changes induced by HPLM (related to Fig.1)

(A) Overlap of quantified proteins across three replicates. More than 10,000 proteins were quantified per replicate, totaling 11,482 proteins. Of those, 9,002 proteins were quantified in all three replicates.

(B) A total of 35,817 phosphopeptides were quantified, with more than 24,000 sites per replicate. Across all three biological replicates, 15,149 sites were consistently quantified.

(C) Correlation heatmap of the TMTpro-scaled protein values for each pair of replicates (54 samples total) from the nine cell lines grown in DMEM and HPLM.

(D) Coefficient of variation (CV) (n = 3) for either DMEM- or HPLM-grown cells is shown.

(E) The UpSet plot illustrates the intersection of proteins that are significantly differentially expressed between HPLM and DMEM culture media, determined by Welch's t-test (S0=variable, 2% FDR – See Experimental Design and Statistical Rationale). The figure displays both the size of the intersection and the count of overlapping hits across all nine cell lines.

(F) Gene ontology analysis (biological process) of proteins significant by Welch's test and consistently changed across six or more cell lines reveals enrichment of cell cycle and metabolic terms.

Figure S3.

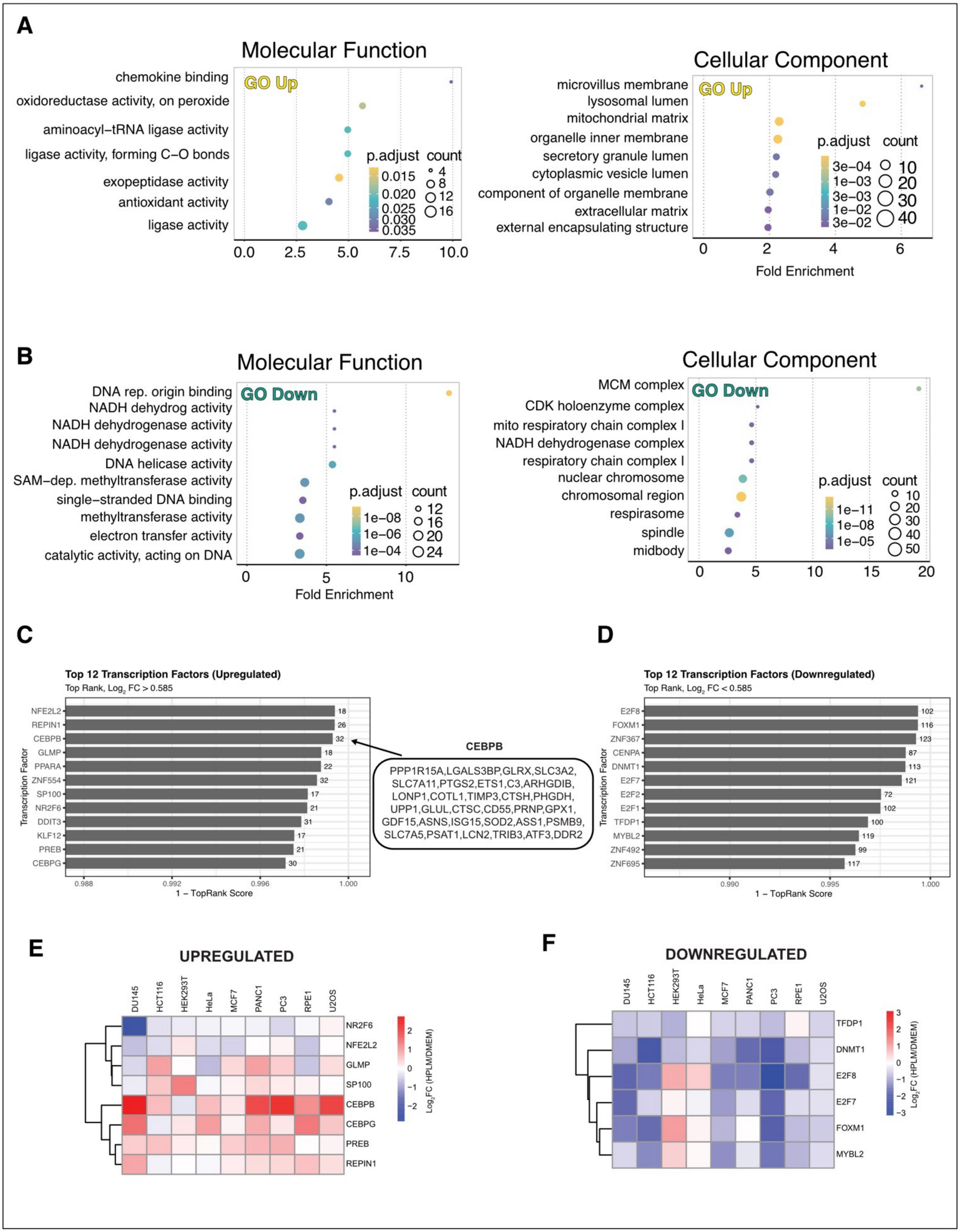
A
Molecular Function
GO Up
chemokine binding
oxidoreductase activity, on peroxide
aminoacyl−tRNA ligase activity
ligase activity, forming C−O bonds
exopeptidase activity
antioxidant activity
ligase activity
Cellular Component
microvillus membrane
lysosomal lumen
mitochondrial matrix
organelle inner membrane
secretory granule lumen
cytoplasmic vesicle lumen
component of organelle membrane
extracellular matrix
external encapsulating structure
Fold Enrichment
B
Molecular Function
GO Down
DNA rep. origin binding
NADH dehydrog activity
NADH dehydrogenase activity
NADH dehydrogenase activity
DNA helicase activity
SAM−dep. methyltransferase activity
single−stranded DNA binding
methyltransferase activity
electron transfer activity
catalytic activity, acting on DNA
Fold Enrichment
Cellular Component
MCM complex
CDK holoenzyme complex
mito respiratory chain complex I
NADH dehydrogenase complex
respiratory chain complex I
nuclear chromosome
chromosomal region
respirasome
spindle
midbody
C
Top 12 Transcription Factors (Upregulated)
Top Rank, Log2 FC > 0.585
NFE2L2, REPIN1, CEBPB, GLMP, PPARA, ZNF554, SP100, NR2F6, DDIT3, KLF12, PREB, CEBPG
Transcription Factor
1 − TopRank Score
CEBPB
PPP1R15A,LGALS3BP,GLRX,SLC3A2, SLC7A11,PTGS2,ETS1,C3,ARHGDIB, LONP1,COTL1,TIMP3,CTSH,PHGDH, UPP1,GLUL,CTSC,CD55,PRNP,GPX1, GDF15,ASNS,ISG15,SOD2,ASS1,PSMB9, SLC7A5,PSAT1,LCN2,TRIB3,ATF3,DDR2
D
Top 12 Transcription Factors (Downregulated)
Top Rank, Log2 FC < 0.585
E2F8, FOXM1, ZNF367, CENPA, DNMT1, E2F7, E2F2, E2F1, TFDP1, MYBL2, ZNF492, ZNF695
E
UPREGULATED
DU145, HCT116, HEK293T, HeLa, MCF7, PANC1, PC3, RPE1, U2OS
NR2F6, NFE2L2, GLMP, SP100, CEBPB, CEBPG, PREB, REPIN1
Log2FC (HPLM/DMEM)
F
DOWNREGULATED
TFDP1, DNMT1, E2F8, E2F7, FOXM1, MYBL2
Log2FC (HPLM/DMEM)

Figure S3. Gene Ontology analysis of proteome changes induced by HPLM (related to Fig.1).

(A,B) Molecular function and cellular component enriched in proteins that are significant in a two-way ANOVA for media effect and are commonly (with a median $\log_2$ fold change of ± 0.585 across all nine cell lines) either upregulated (A) or downregulated (B) upon HPLM culture.

(C,D) Transcription factor enrichment analysis was conducted with ChEA3 on proteins that showed significant differential expression, contributing to HPLM-driven differences, identified by ANOVA (FDR-corrected). These proteins were either upregulated (C) or downregulated (D), with a minimum $\log_2$ fold change of 0.585. Gene targets contributing to CEBPB enrichment are indicated

(E,F). Protein expression levels of candidates identified in C, D after HPLM culture.

Figure S4.

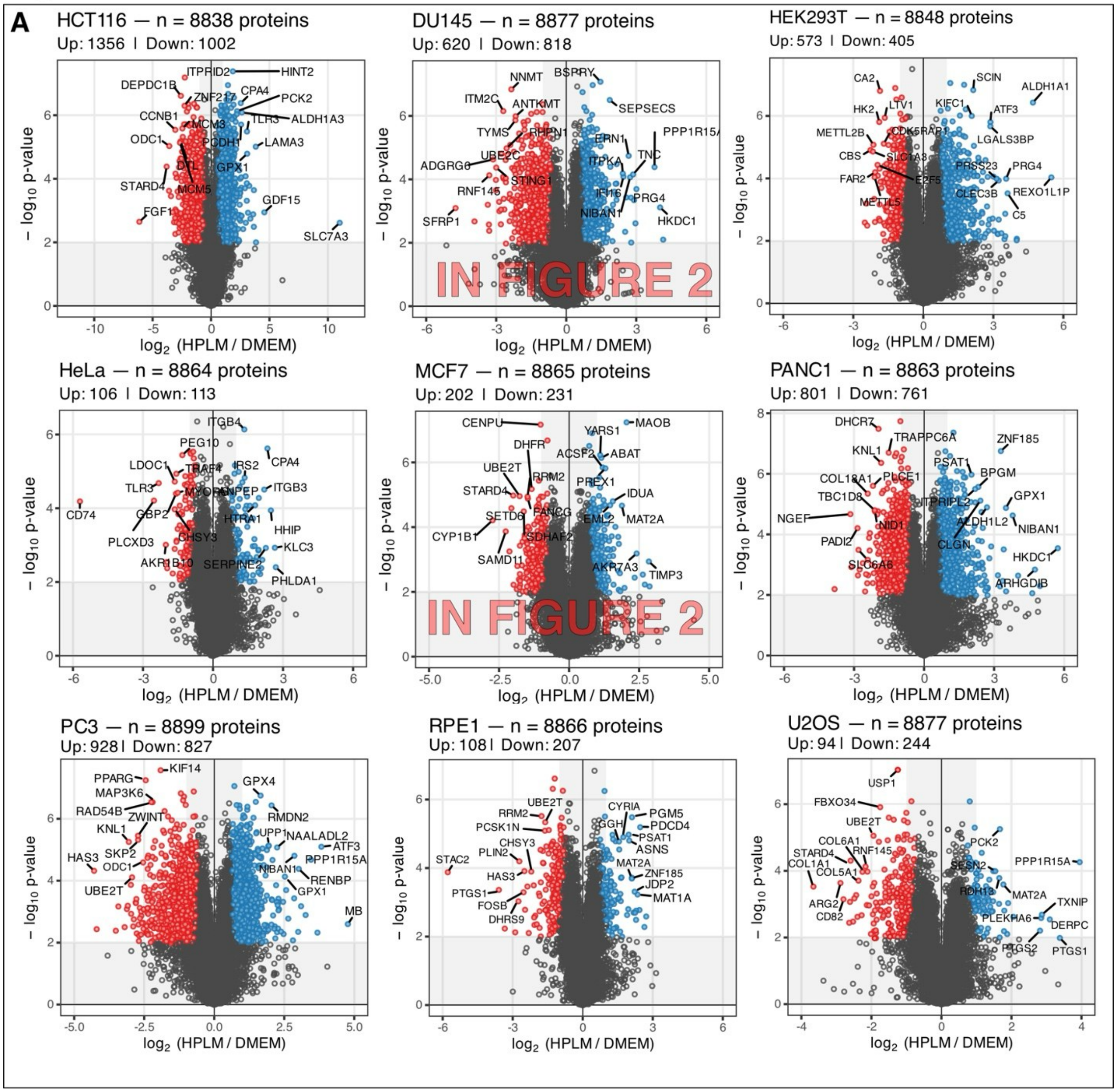

Figure S4. Volcano plots of nine cell line proteomics (related to Fig. 2).

(A) Volcano plots of the -$\log_{10}$-transformed p-value versus the $\log_2$-transformed ratio of HPLM/DMEM conditions for all nine cell lines. P-values were determined using a two-sided Welch's t-test, with a 2% FDR for multiple comparisons. S0 was set to 0.585 for DU145, HCT116, HEK293T, PANC1, and PC3; and to 0.39 for HeLa, MCF7, RPE1, and U2OS.. Only points with p-value of 0.01 or lower were considered significant. Among the statistically significant hits, proteins that are significantly upregulated are circled in blue, while those that are downregulated are in red. n = 3 biological replicates.

Figure S5.

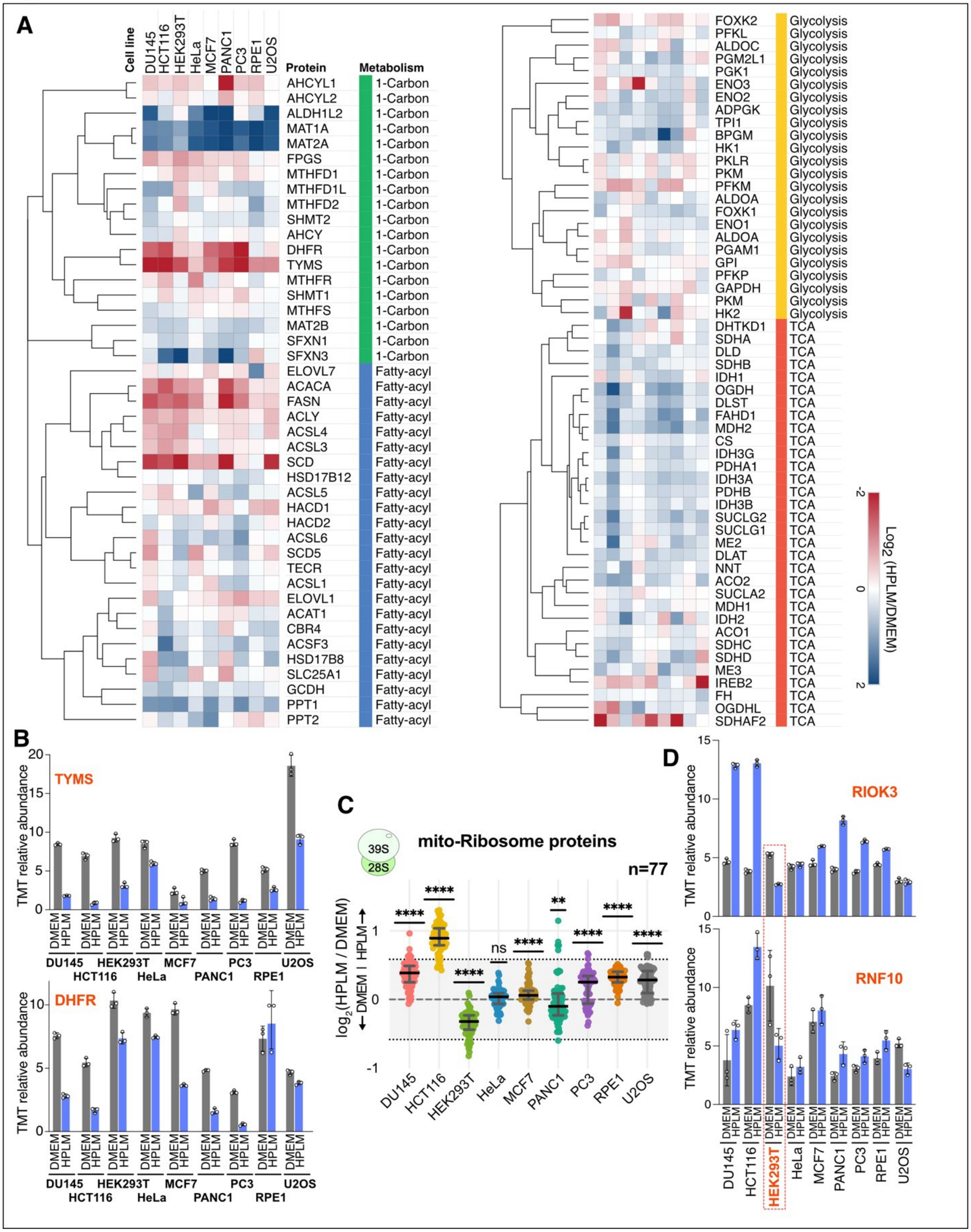

Figure S5. Metabolic proteome response to HPLM (related to Fig. 2).

(A) Heatmap of 90 metabolic enzymes in the 1-Carbon, Fatty-acyl, Glycolysis, and tricarboxylic acid cycle (TCA) pathways is shown. The mean of the three biological replicates is used for the heatmap.
(B) Relative abundance (TMT-scaled value) of TYMS (top) and DHFR (bottom) is shown as bar graphs. Mean ± S.D. for three biological replicates.
(C) The scatter plot shows the Log_2 ratio of HPLM/DMEM for mitochondrial ribosomal proteins across the 9 cell lines. Thick black lines represent the median ratio values (± interquartile range) of 77 representative proteins (n = 3 biological triplicate experiments). p-values were calculated using a Wilcoxon signed-rank test to determine whether the differences between the two culture media were statistically significant ($\alpha = 0.01$). The grey shaded area between the dotted lines indicates a ±1.5 ratio change.
(D) Relative abundance (TMT-scaled value) of iRQC proteins, RIOK3 (top) and RNF10 (bottom), is shown as bar graphs. Mean ± S.D. for three biological replicates. Only HEK293T cells show significant downregulation of these iRQC factors.

Figure S6.

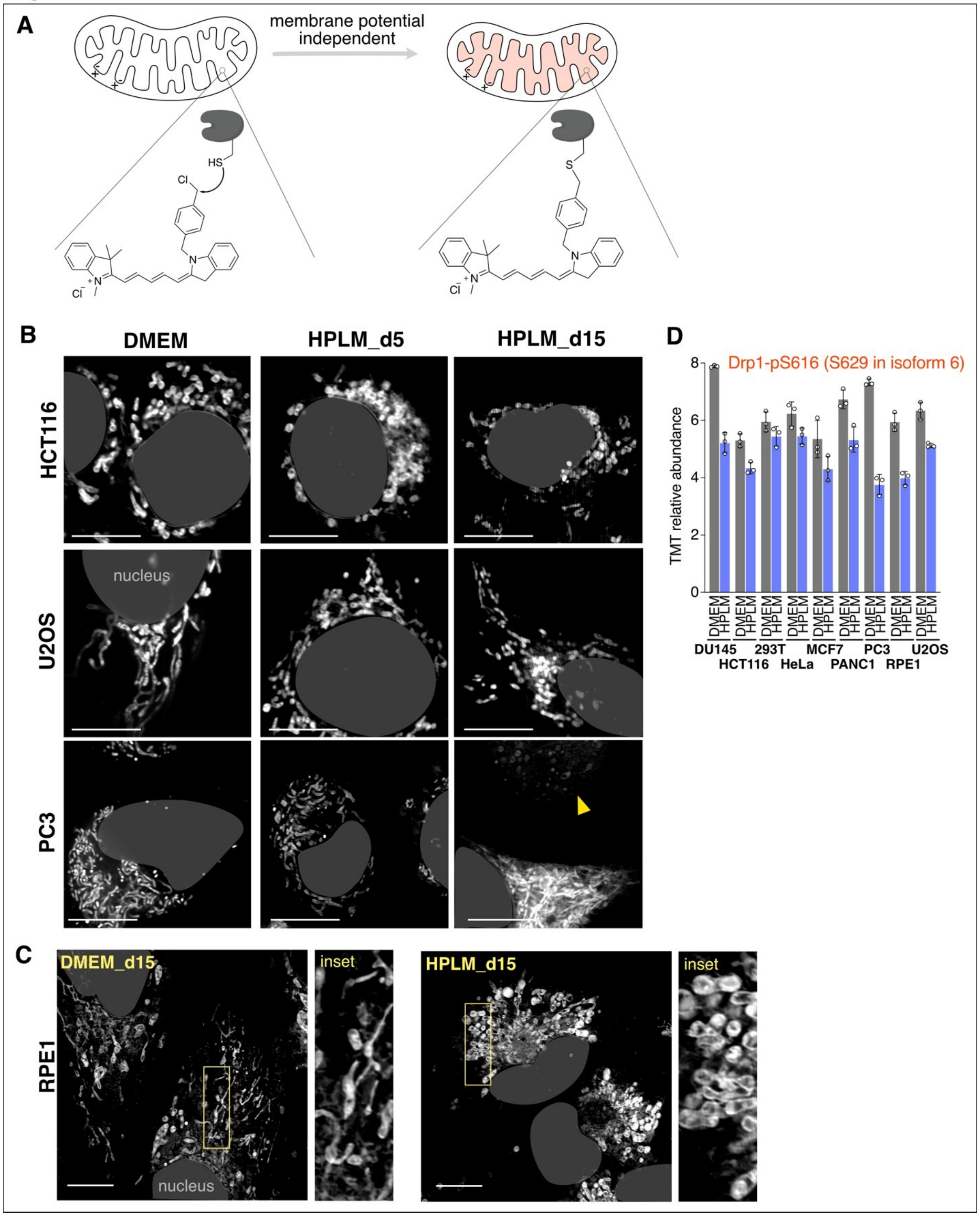

A
membrane potential independent
HS
Cl
S
N
Cl⁻
B
DMEM
HPLM_d5
HPLM_d15
HCT116
U2OS
PC3
nucleus
C
RPE1
DMEM_d15
HPLM_d15
inset
nucleus
D
Drp1-pS616 (S629 in isoform 6)
TMT relative abundance
0
2
4
6
8
DMEM
HPLM
DU145
HCT116
293T
HeLa
MCF7
PANC1
PC3
RPE1
U2OS

Figure S6. Effect of HPLM on mitochondria morphology (related to Fig. 3).
(A) Structure of MitoTracker Deep Red, which forms a covalent bond with mitochondrial proteins.
(B) Live imaging of mitochondria in HCT116, U2OS, and PC3 cells treated with DMEM or HPLM for an extended period using MitoTracker Deep Red. Scale bar: 10 µm.
(C) Mitochondria in RPE1 cells exhibit different morphologies after 15 days of culture in DMEM and HPLM, as shown in Fig. 3A. Scale bar: 10 µm.
(D) TMT quantification of DNM1L S616 phosphorylation across 9 cell lines.

Figure S7.

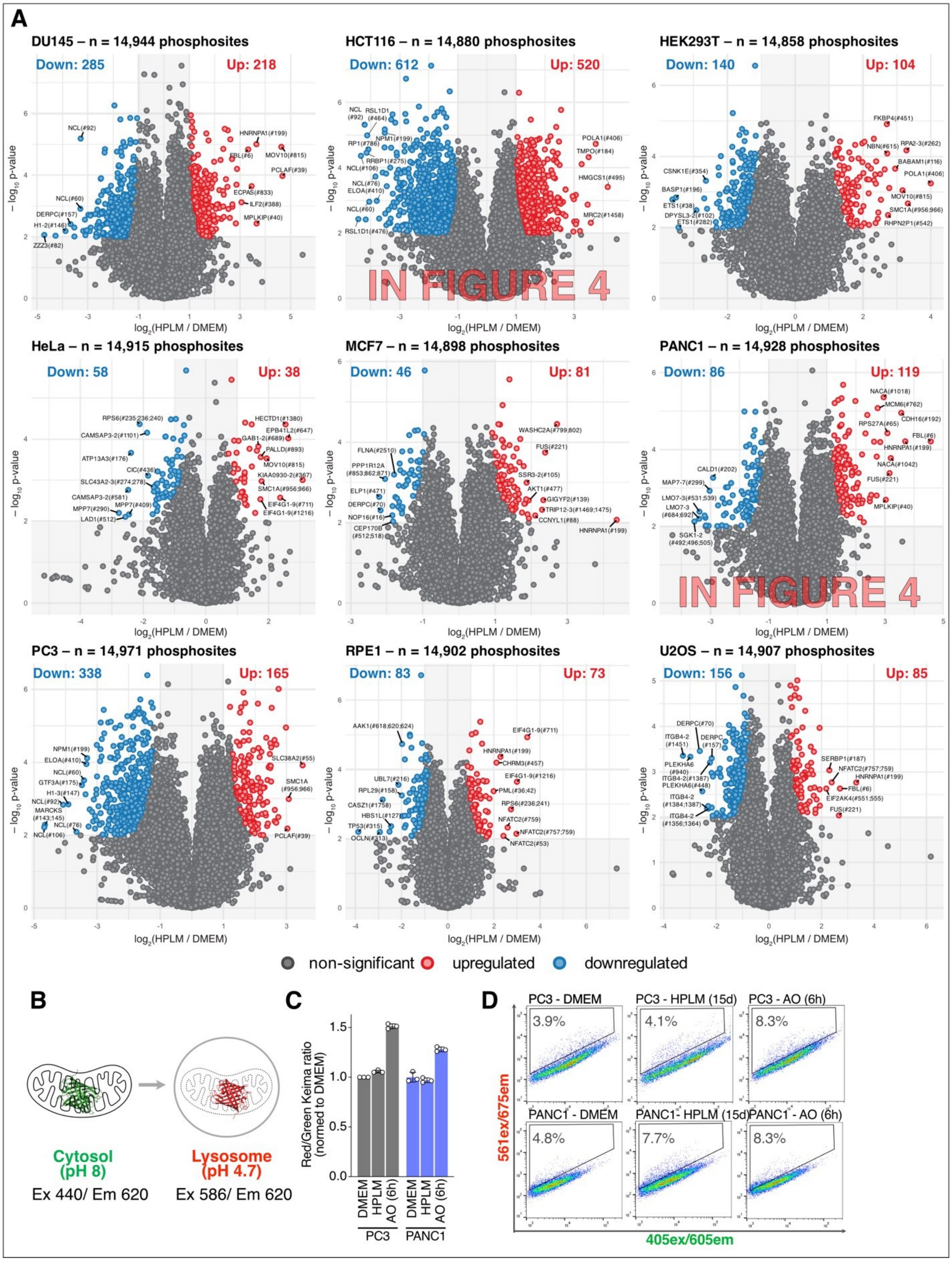

A
DU145 – n = 14,944 phosphosites
Down: 285
Up: 218
HCT116 – n = 14,880 phosphosites
Down: 612
Up: 520
IN FIGURE 4
HEK293T – n = 14,858 phosphosites
Down: 140
Up: 104
HeLa – n = 14,915 phosphosites
Down: 58
Up: 38
MCF7 – n = 14,898 phosphosites
Down: 46
Up: 81
PANC1 – n = 14,928 phosphosites
Down: 86
Up: 119
IN FIGURE 4
PC3 – n = 14,971 phosphosites
Down: 338
Up: 165
RPE1 – n = 14,902 phosphosites
Down: 83
Up: 73
U2OS – n = 14,907 phosphosites
Down: 156
Up: 85
– log10 p-value
log2(HPLM / DMEM)
non-significant
upregulated
downregulated
B
Cytosol
(pH 8)
Ex 440/ Em 620
Lysosome
(pH 4.7)
Ex 586/ Em 620
C
Red/Green Keima ratio (normed to DMEM)
DMEM
HPLM
AO (6h)
PC3
PANC1
D
PC3 - DMEM
3.9%
PC3 - HPLM (15d)
4.1%
PC3 - AO (6h)
8.3%
PANC1 - DMEM
4.8%
PANC1- HPLM (15d)
7.7%
PANC1 - AO (6h)
8.3%
561ex/675em
405ex/605em

Figure S7. Phosphoproteome remodeling upon HPLM culture across cell lines (related to Fig. 4).

(A) Analysis of the phosphoproteomic data is shown as a volcano plot of the -$\log_{10}$-transformed p-value versus the $\log_2$-transformed ratio of HPLM/DMEM conditions for the nine cell lines. Each point represents a peptide containing the phosphorylation site. P-values were calculated using a two-sided Welch's t-test (adjusted to 1% FDR for multiple comparisons, S0 = 0.585 for DU145, HCT116, and PC3, 2% FDR and S0 = 0.585 for HEK293T and PANC1, 3.5% FDR and S0 = 0.15 for HeLa, 2% FDR and S0 = 0.3 for MCF7, RPE1, and U2OS). Among the statistically significant hits, sites that are significantly upregulated are circled in red, while those that are downregulated are in blue. Only values with p-value of 0.01 or lower were considered significant. The total number of sites identified in all 3 MS runs per cell line are indicated at the top of each volcano. n = 3 biological replicates.

(B) Schematic of the mito-Keima reporter.

(C) Average Red/Green Keima ratio from over 2000 cells measured under the specified condition is shown as mean ± SD.

(D) Density plot of cells expressing the mito-Keima reporter.

Figure S8.

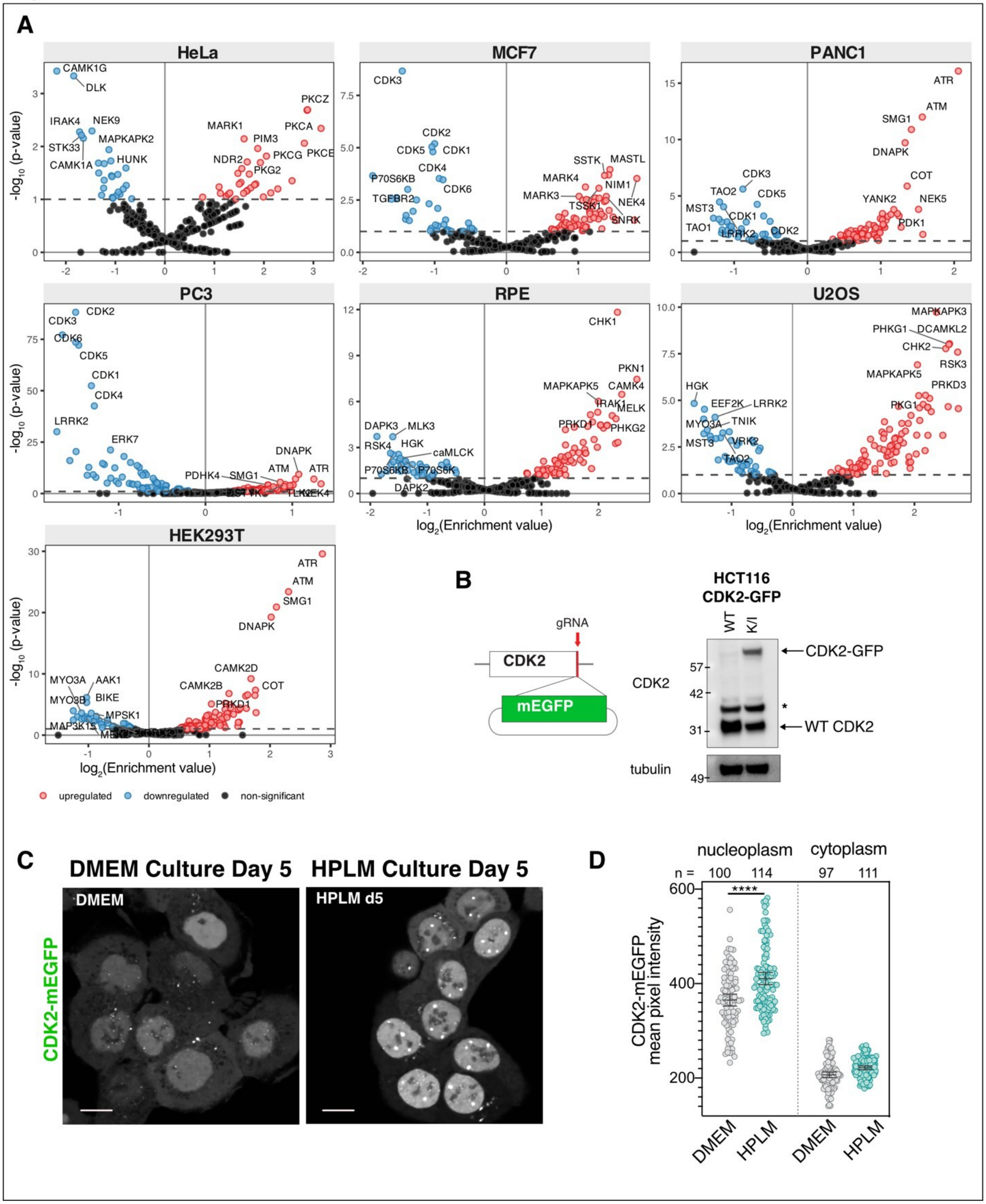
A
HeLa
MCF7
PANC1
PC3
RPE
U2OS
HEK293T
-log10 (p-value)
log2(Enrichment value)
upregulated
downregulated
non-significant
B
HCT116 CDK2-GFP
WT
KI
gRNA
CDK2
mEGFP
CDK2-GFP
WT CDK2
tubulin
C
DMEM Culture Day 5
HPLM Culture Day 5
DMEM
HPLM d5
CDK2-mEGFP
D
nucleoplasm
cytoplasm
n = 100 114 97 111
****
CDK2-mEGFP mean pixel intensity
DMEM
HPLM

Figure S8. Kinase motif enrichment analysis reveals a decrease in CDKs activity as a common feature of HPLM-cultured cells (related to Fig. 5).

(A) Motif enrichment analysis to identify kinase-specific signatures from phosphoproteomics data across seven cell lines.

(B) Immunoblot analysis of HCT116 engineered by CRISPR-Cas9 shows heterozygous knock-in of CDK2 with mEGFP at the C-terminus. Asterisk: nonspecific band.

(C) Live imaging analysis of HCT116 cells endogenously expressing CDK2-mEGFP was performed to examine the subcellular localization of CDK2 following a five-day culture in the specified media. Scale bar: 10 µm.

(D) The mean pixel intensity of individual cells imaged as in panel C was quantified and plotted with mean ± S.E.M. n = 100, 114, 97, 111 cells quantified.

Figure S9.

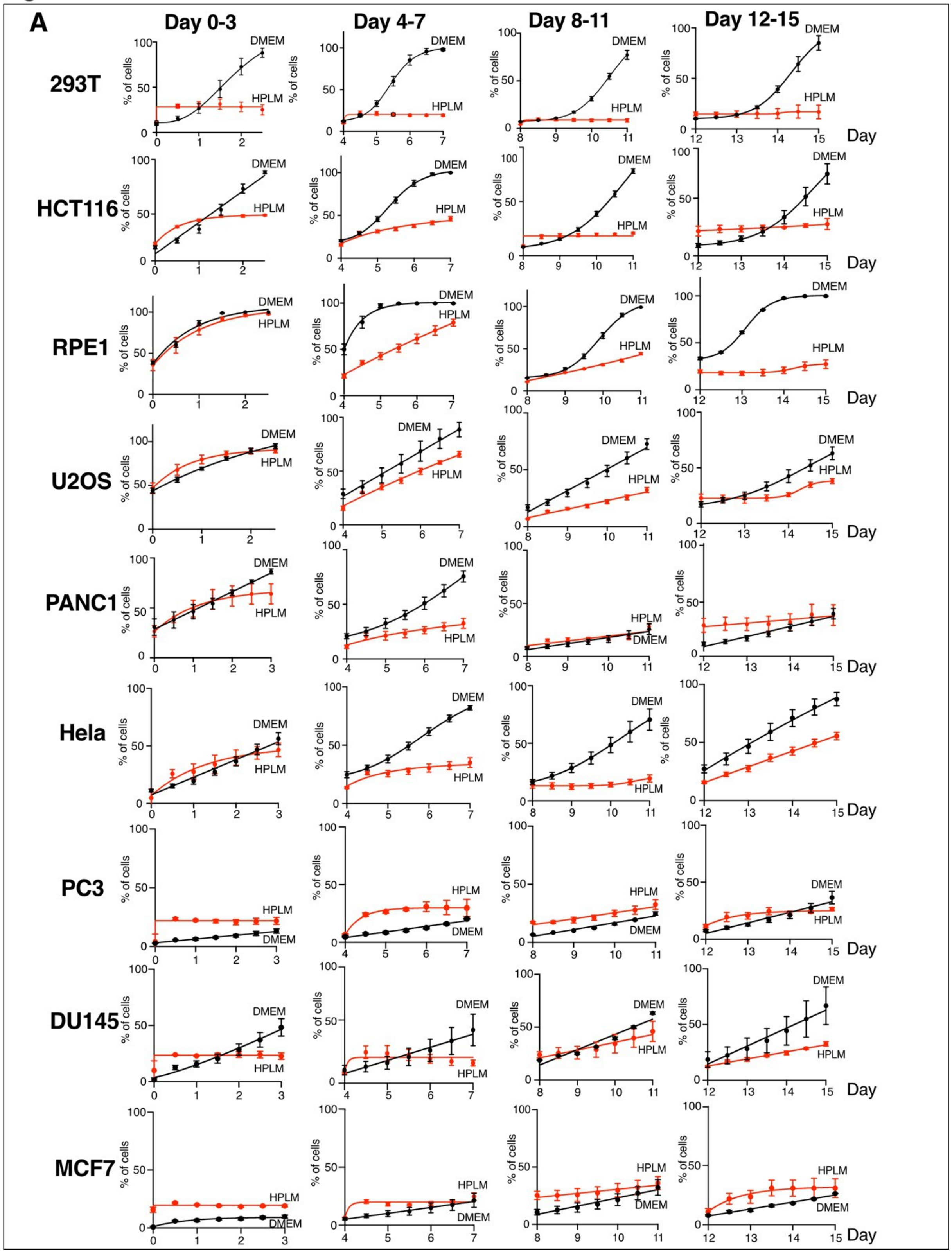
A
Day 0-3
Day 4-7
Day 8-11
Day 12-15
293T
HCT116
RPE1
U2OS
PANC1
Hela
PC3
DU145
MCF7
DMEM
HPLM
% of cells
Day

Figure S9. Cell proliferation assay for nine cell lines. (related to Fig. 5).
(A) The nine cell lines were cultured in DMEM and HPLM, and proliferation was measured using IncuCyte at the indicated times over a 15-day period.
